# Supplementary figures and images for: Exploring the potential of structure-based deep learning approaches for T cell receptor design
Source: PLoS Comput Biol. 2024 Sep 30;20(9):e1012489. doi: 10.1371/journal.pcbi.1012489 (PMC11466415; doi:10.1371/journal.pcbi.1012489)

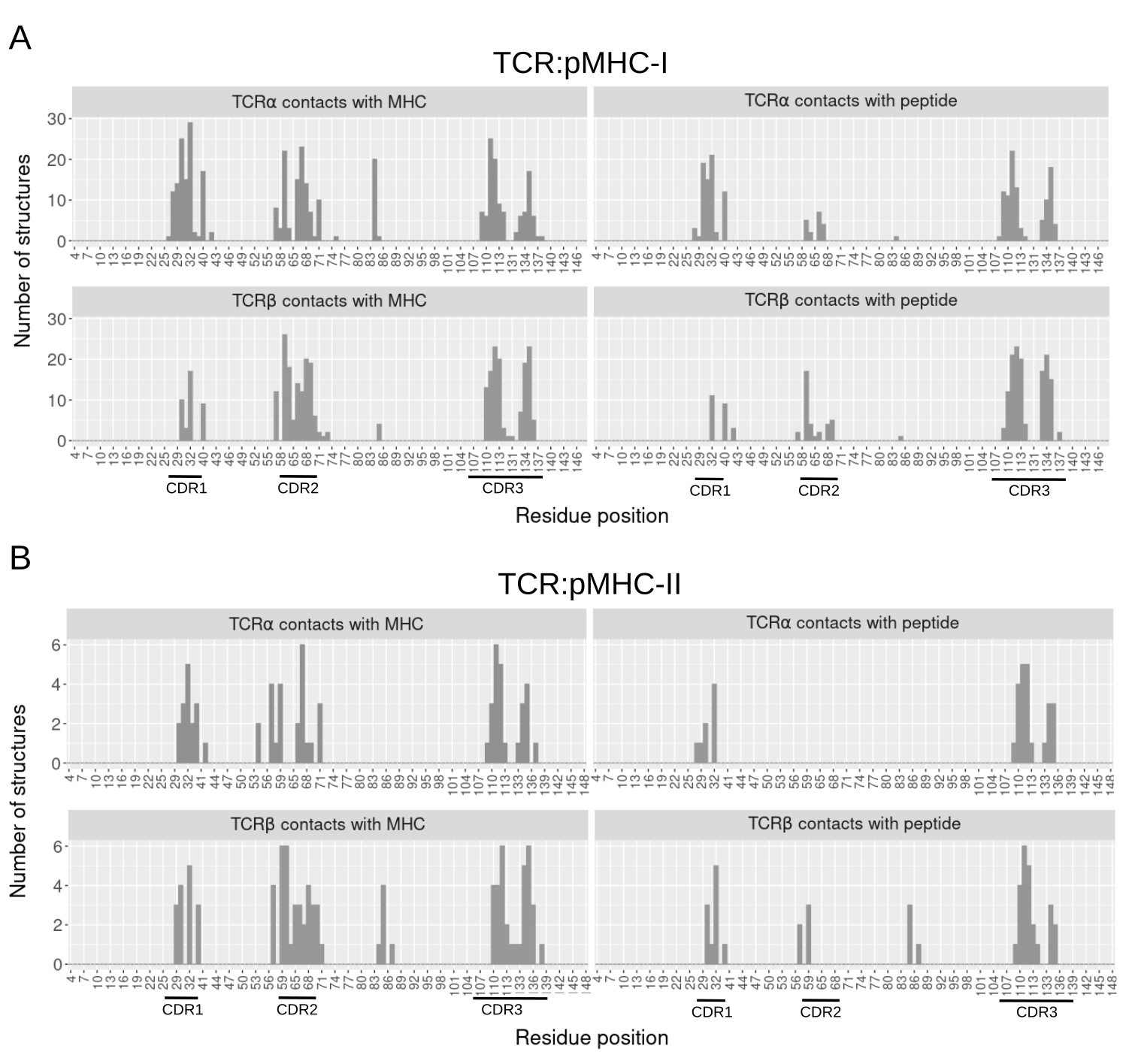

Supplement: S1 Fig — (A) The upper panels show contacts between the TCRα variable domain and the peptide or MHC-I, while the lower panels display contacts between the TCRβ variable domain and the peptide or MHC. The left panels detail TCRα and TCRβ contacts with the MHC-I, and the right panels show TCRα and TCRβ contacts with the peptide. Contacts are identified by TCR position, using the Aho numbering scheme, with CDR positions indicated. The y-axis represents the number of structures (total of 32 in the MHC-I test set) where each contact is observed. (B) same as (A) but considering contacts with the peptide and MHC in the MHC-II test set (total of 6 structures). (TIF) [file pcbi.1012489.s001.tif]

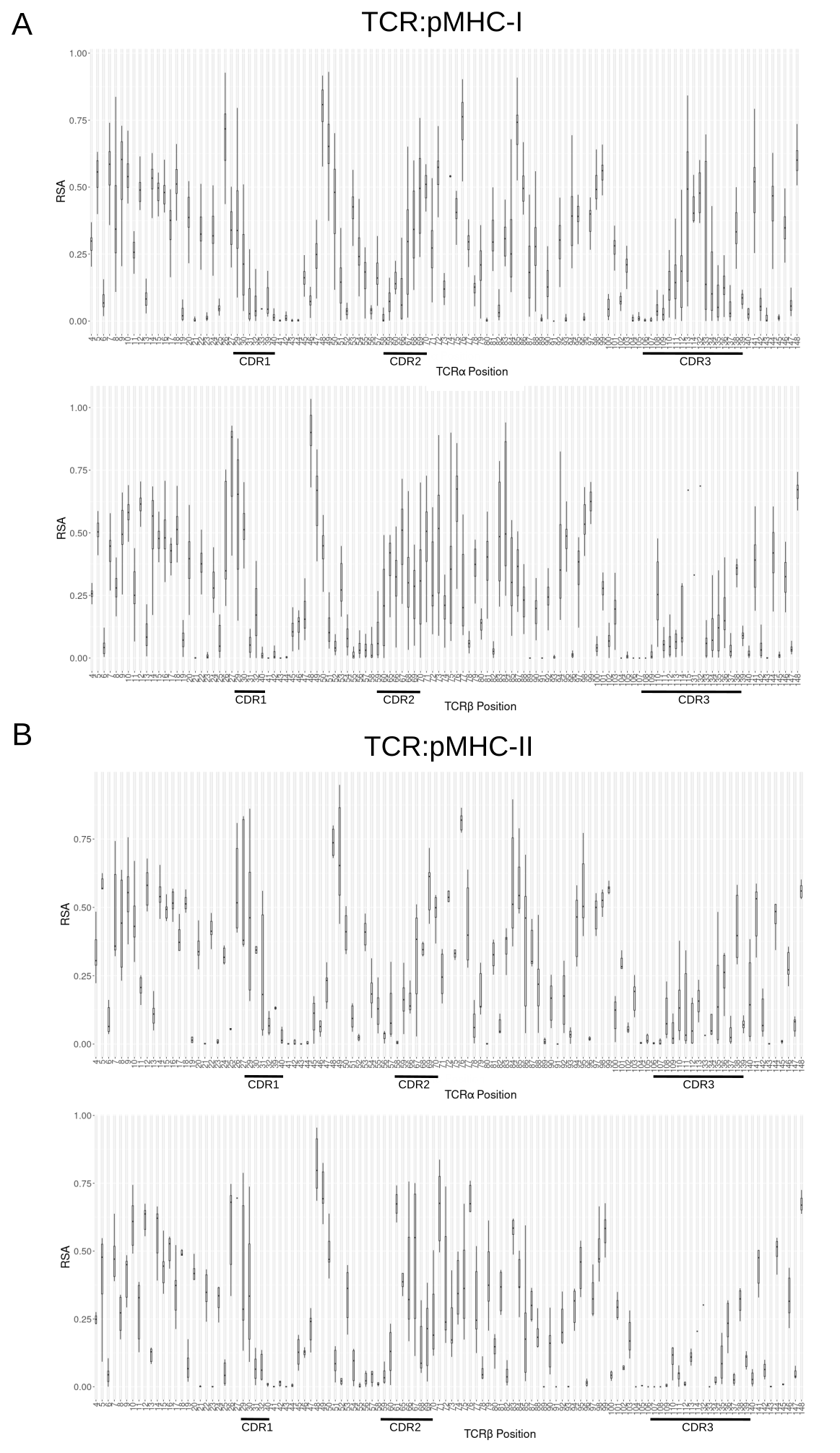

Supplement: S2 Fig — (A) Box plot of the RSA for TCRα positions (upper panel) and TCRβ positions (lower panel) considering the TCRs from the complex bound with pMHC-I (a total of 32 complexes in the test set). (B) same as (A) but considering the TCR from the set of MHC-II bound complexes. CDR positions are indicated. (TIF) [file pcbi.1012489.s002.tif]

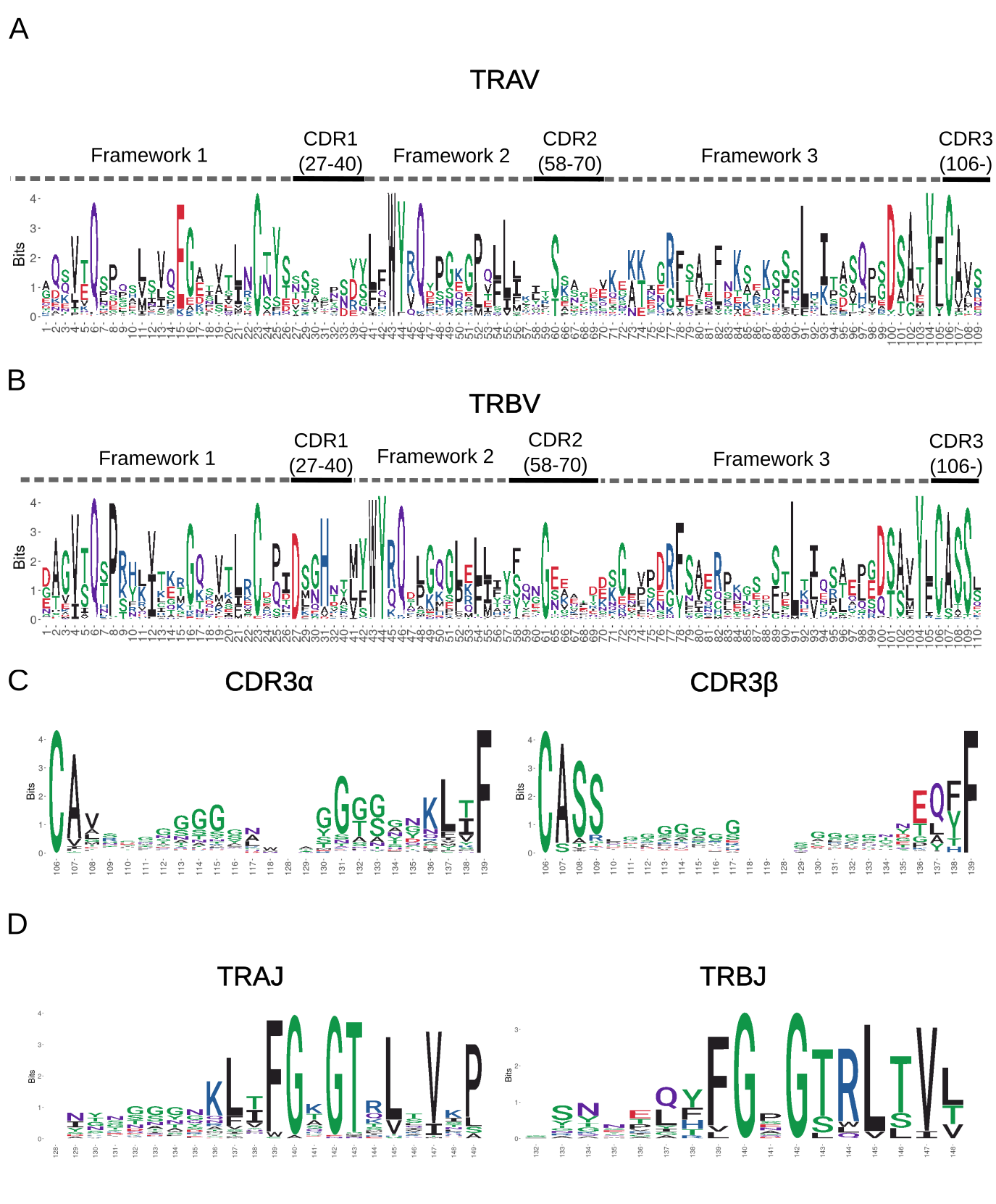

Supplement: S3 Fig — (A) Sequence variability across the TRAV domain. The sequences are numbered according to Aho numbering scheme. Sequence gaps consistently observed at the same position in all sequences were removed from the logos for clarity. See the Methods section for details on the construction and processing of the sequence set. (B) Same as (A), but for TRBV domain. (C) Sequence variability across human CDR3α (on the left) and CDR3β (on the right) sequences from TCRs with known antigen specificity. (D) Sequence variability across TRAJ (on the left) and TRBJ (on the right). TRAJ and TRBJ numbering were assigned based on the CDR3 C-terminus as reference. (TIF) [file pcbi.1012489.s003.tif]

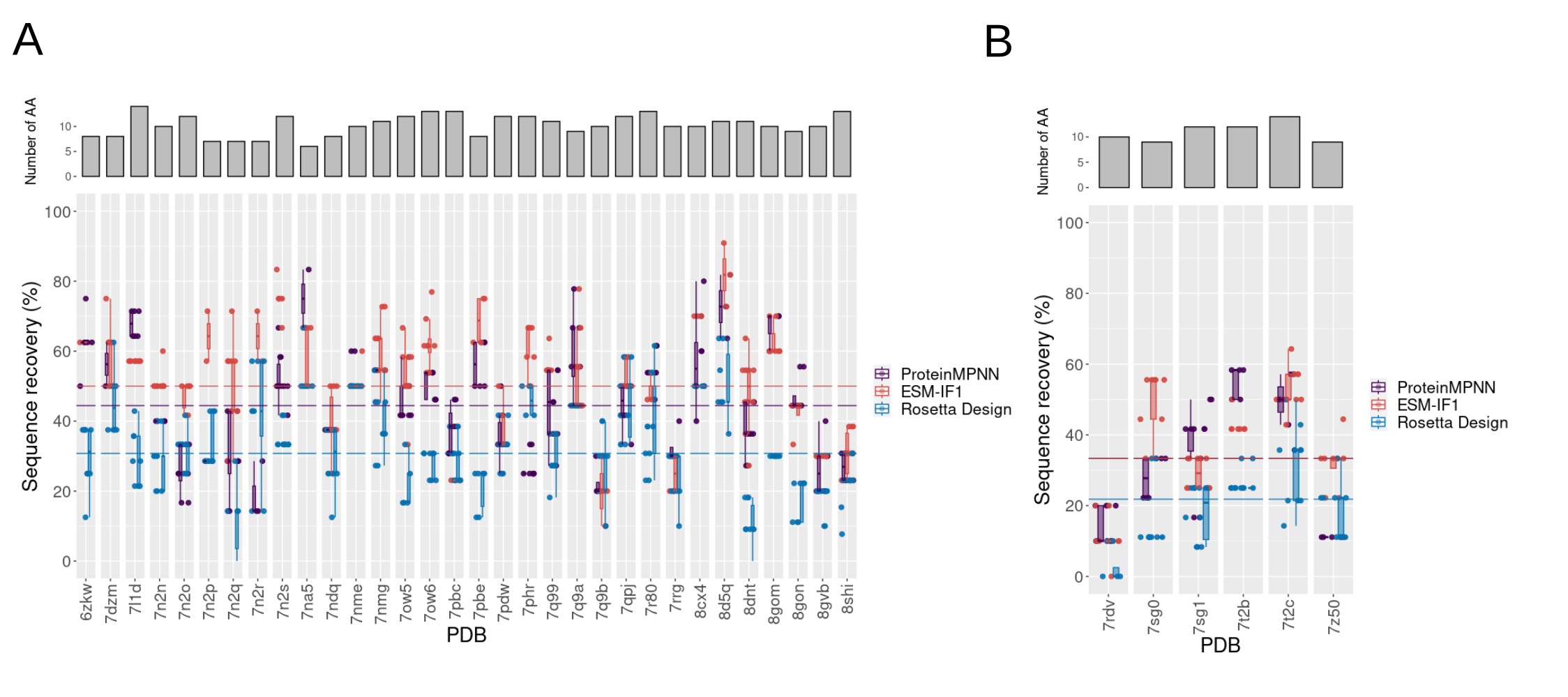

Supplement: S4 Fig — (A) Upper panel shows the number of interface amino acids selected to be designed for each test case. Bottom panel presents boxplots of sequence recovery for each case designed by ProteinMPNN (purple), ESM-IF1 (red) or Rosetta (blue). Each point corresponds to a design sequence. Redundant designs were removed from the analysis. For both methods, 10 designs were generated per test cases and for Rosetta. Lines indicate the median computed over all designs: 44.4%, 50.0% and 30.8% for ProteinMPNN, ESM-IF1, and Rosetta Design, respectively. (B) Same as (A), but for MHC-II. Lines are median over all designs: 33.3%, 33.3%, and 21.8% for ProteinMPNN, ESM-IF1 and Rosetta Design, respectively. (TIF) [file pcbi.1012489.s004.tif]

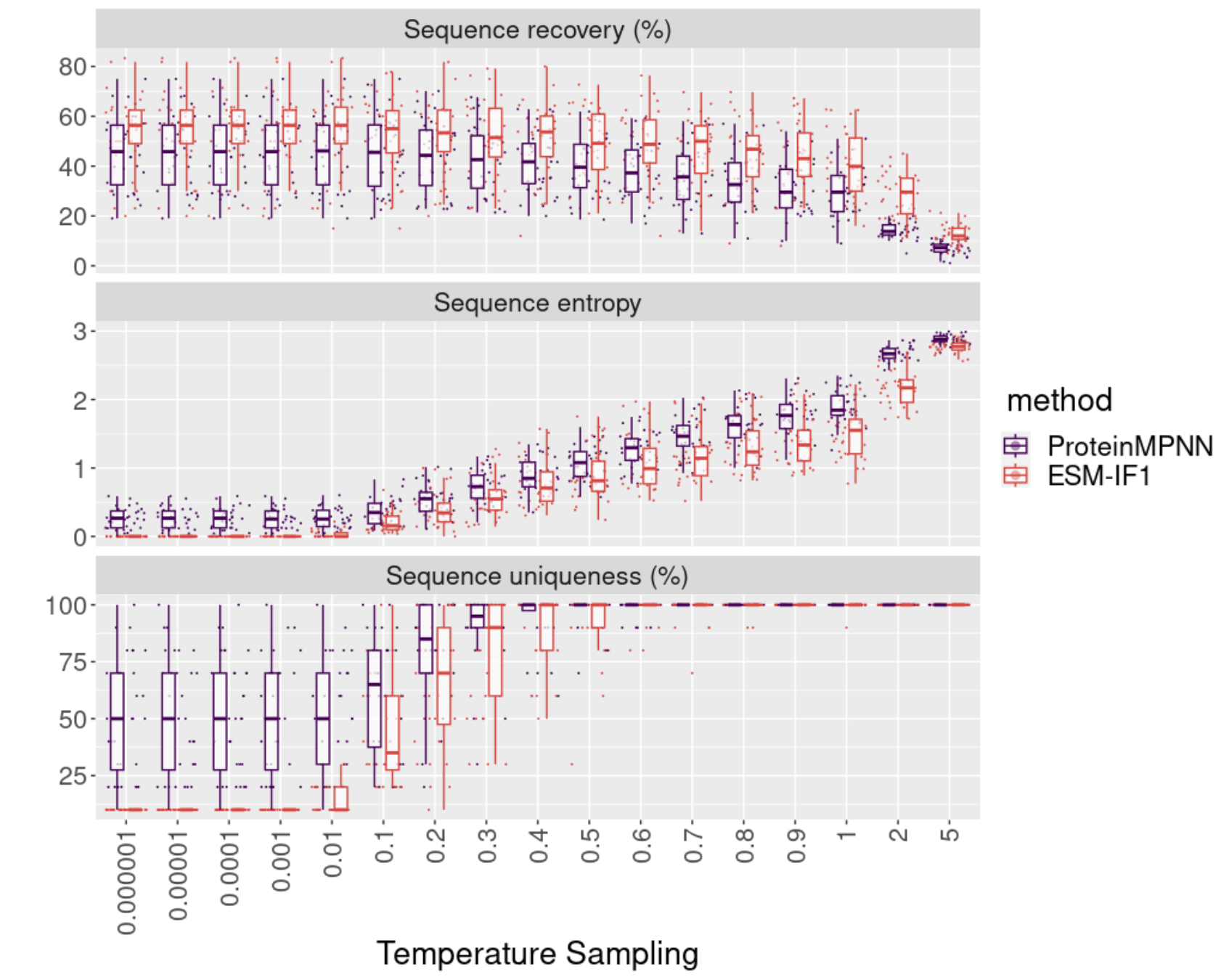

Supplement: S5 Fig — The upper panel presents the sequence recovery relative to the temperature sampling. Each point corresponds to the average sequence recovery of each MHC-I test case. The middle panel presents the entropy of the designed sequences for each MHC-I test case in function of the temperature sampling. Higher entropy indicates higher diversity in the generated sequences. The entropy was calculated using the R Bio3D package as an average of positional entropies. The bottom panel shows the uniqueness of generated sequences in function of temperature. Maximum uniqueness (100%) indicates that all generated sequences for a given test case are different. The tested temperatures ranged from 0.000001 to 5. (TIF) [file pcbi.1012489.s005.tif]

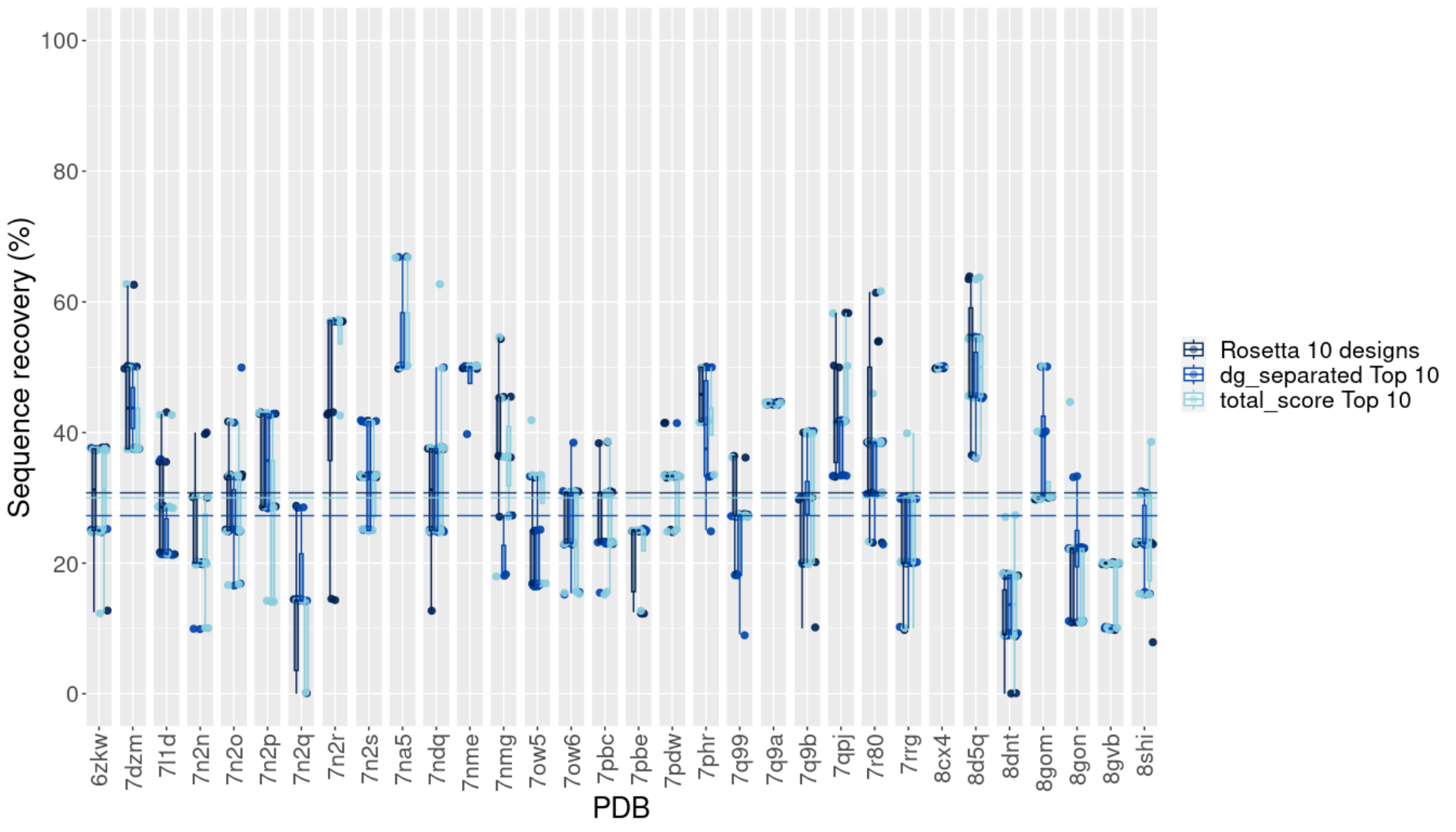

Supplement: S6 Fig — Dark blue box plots represent the sequence recovery for each target using the default Rosetta InterfaceDesign2019 protocol and generating 10 designs per target. The medium blue box plots represent the sequence recovery of top 10 designs scored by Rosetta dG_separated term from a total of 1000 generated designs. The light blue box plots represent the sequence recovery of top 10 design scores by Rosetta total_score term from a total of 1000 generated designs. Each point corresponds to a design sequence. Redundant designs were removed from the analysis. Lines indicate the median computed over all designs: 30.8%, 27.3%, and 30.0% for 10 designs with default Rosetta protocol, top 10 best dG_separated score designs and top 10 best total_score scored designs, respectively. (TIF) [file pcbi.1012489.s006.tif]

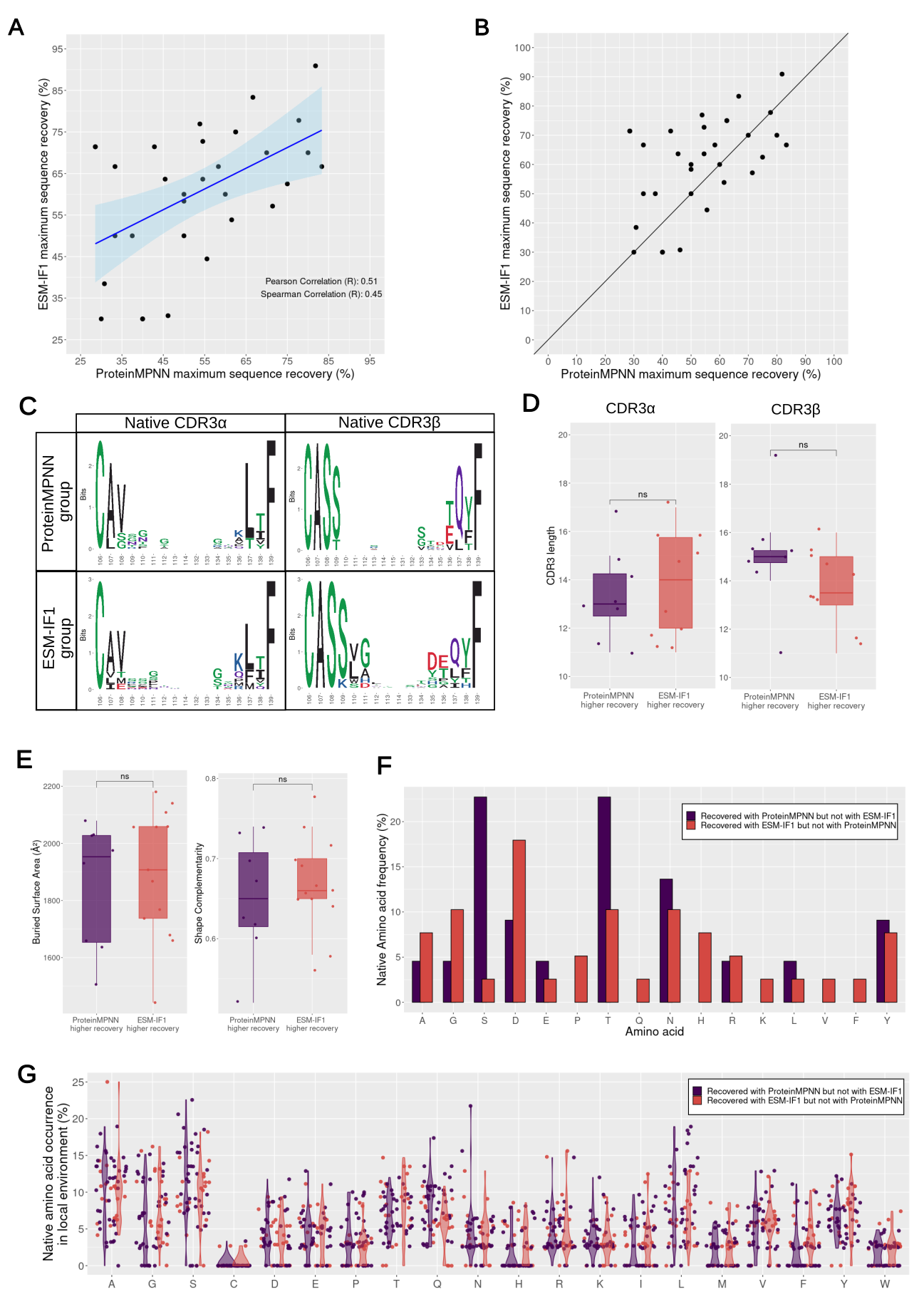

Supplement: S7 Fig — (A) Scatter plot with a linear trend line and confidence interval of 0.95 (light blue region) presenting the correlation between the maximum sequence recovery of ProteinMPNN and ESM-IF1. Correlation coefficients are indicated in the plot and each point representing an MHC-I test case. (B) Scatter plot comparing the maximum sequence recovery of ProteinMPNN and ESM-IF1 with a diagonal line to aid in visual comparison. All designs were obtained with T = 0.1 for ProteinMPNN and T = 0.2 for ESM-IF1. (C), (D) and (E) panels present the comparison between test cases where ProteinMPNN achieved a higher maximum sequence recovery (at least 10% higher) than ESM-IF1 (6zkw, 7l1d, 7na5, 7pbc, 7rrg, 8cx4, 8gon, and 8gvb test cases in ProteinMPNN group) and test cases where ESM-IF1 achieved a higher maximum sequence recovery (at least 10% higher) than ProteinMPNN (7dzm, 7n2n, 7n2o, 7n2p, 7n2q, 7n2r, 7n2s, 7ndq, 7nmg, 7ow6, 7pbe, 7phr, and 8dnt test cases in ESM-IF1 group). (C) Sequence logos of CDR3α and CDR3β from test cases where ProteinMPNN achieved a higher maximum sequence recovery than ESM-IF1 (ProteinMPNN group) are shown in the first row. In the second row, sequence logos are presented for test cases where ESM-IF1 achieved a higher maximum sequence recovery than ProteinMPNN (ESM-IF1 group). The sequences are numbered according to the Aho numbering scheme. Only non-redundant CDR3 sequences (8 for the ProteinMPNN group and 10 for ESM-IF1 group) were considered in the sequence logo and only positions observed in at least one sequence are presented. (D) Box plots showing the length distribution of CDR3α (left) and CDR3β (right) from test cases where ProteinMPNN achieved a higher maximum sequence recovery than ESM-IF1 (in purple) and from test cases where ESM-IF1 outperformed ProteinMPNN (in red). Only non-redundant CDR3 are presented. (E) Box plots of the Buried Surface Area (in Å2) (left) and Shape Complementarity (right) of the interface between the TCR and pMHC, obtained fro [file pcbi.1012489.s007.tif]

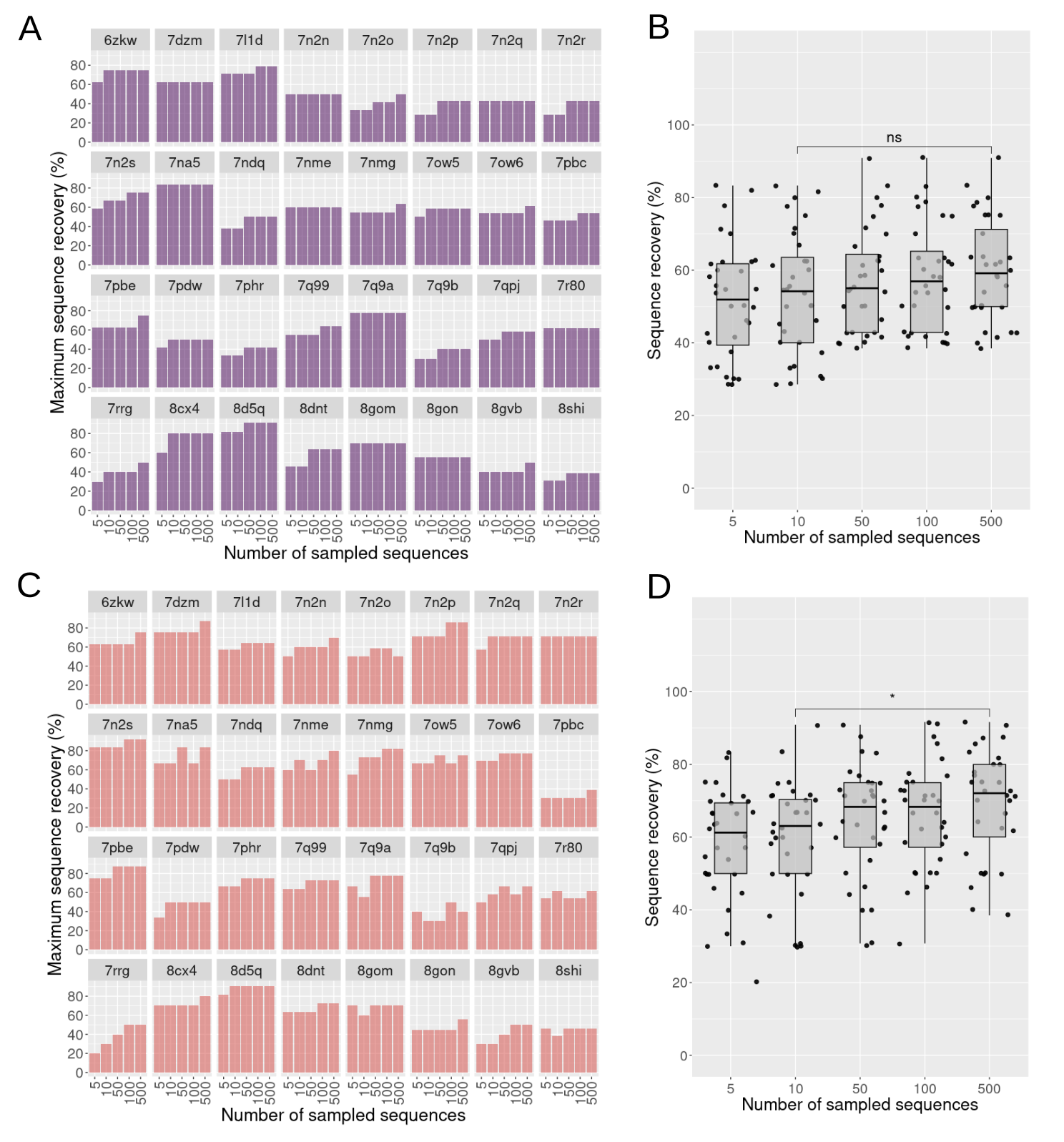

Supplement: S8 Fig — (A) Effect of increasing number of ProteinMPNN generated designs (from 5 to 500 generated designs) with T = 0.1 on the maximum sequence recovery for each MHC-I test case. (B) Box plot of the maximum sequence recovery per number of generated sequences by ProteinMPNN. Each point corresponds to the maximum sequence recovery observed at the given number of sampled sequences of a given test case. (C) Same as (A), but with ESM-IF1. (D) Same as (B), but with ESM-IF1. Statistical pairwise comparison between sampling of 10 and 500 using Mann-Whitney test with the R ggpubr package. Significance is indicated above each box plot (* corresponds to a p- value ≥ 0.05 and, while ‘ns’ means no significance (p- value > 0.5)). (TIF) [file pcbi.1012489.s008.tif]

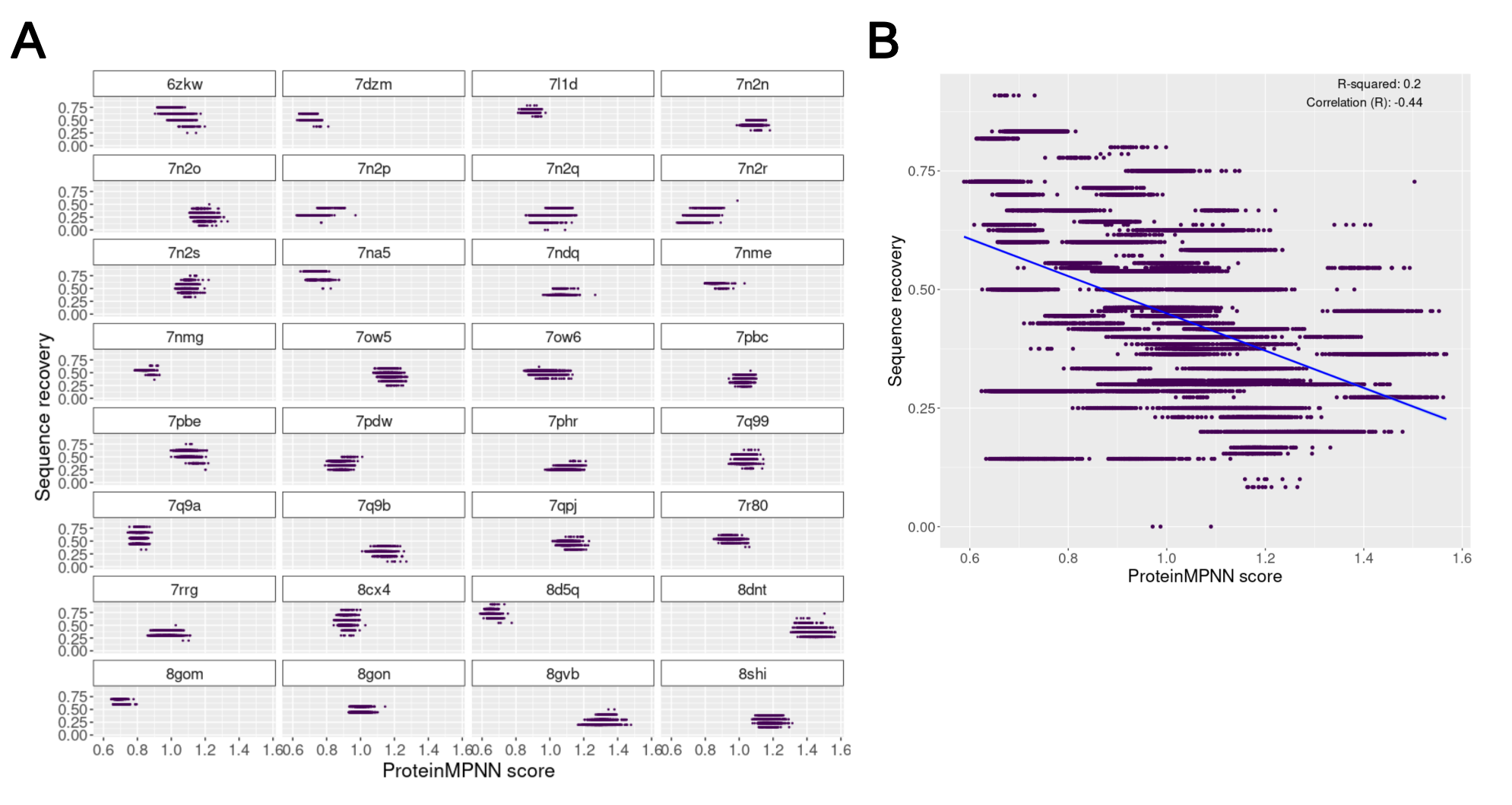

Supplement: S9 Fig — (A) Scatter plot of sequence recovery and ProteinMPNN scores from 1000 ProteinMPNN designs for each test case. Redundant sequences were not removed in this analysis since the same sequence can have different ProteinMPNN scores. (B) Scatter plot considering all designs together with a linear trend line (blue line) presenting the correlation between the sequence recovery and ProteinMPNN score. Correlation coefficients are indicated in the plot. The correlation coefficient was determined using Spearman. (TIF) [file pcbi.1012489.s009.tif]

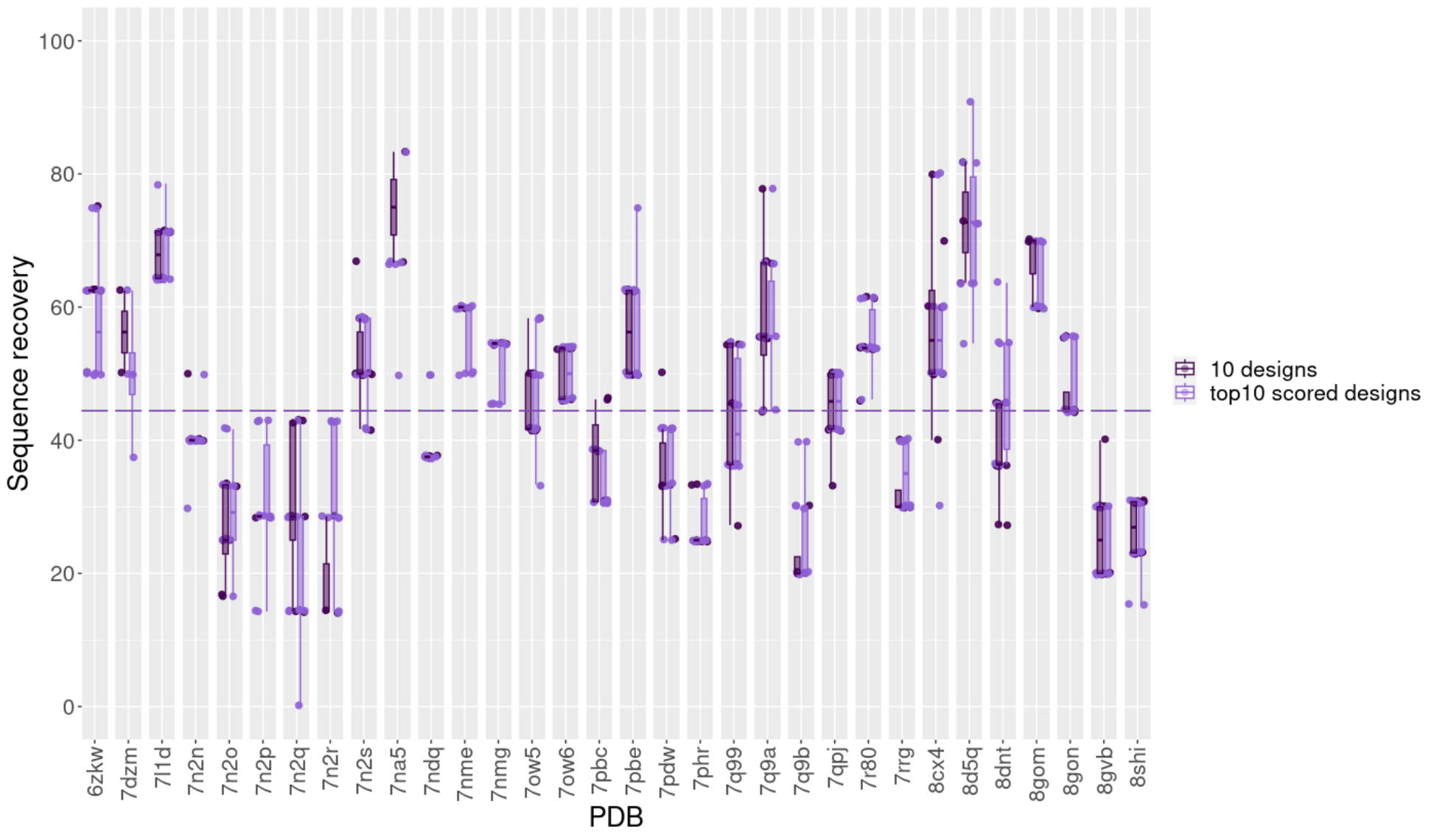

Supplement: S10 Fig — The box plots present the sequence recovery for the 10 ProteinMPNN designs (in dark purple) and for the top 10 scored designs (in light purple). Each point corresponds to the sequence recovery of a designed sequence. Redundant sequences within each test case were excluded from the analysis. Lines indicate the median computed over all designs: 44.4% for both cases. (TIF) [file pcbi.1012489.s010.tif]

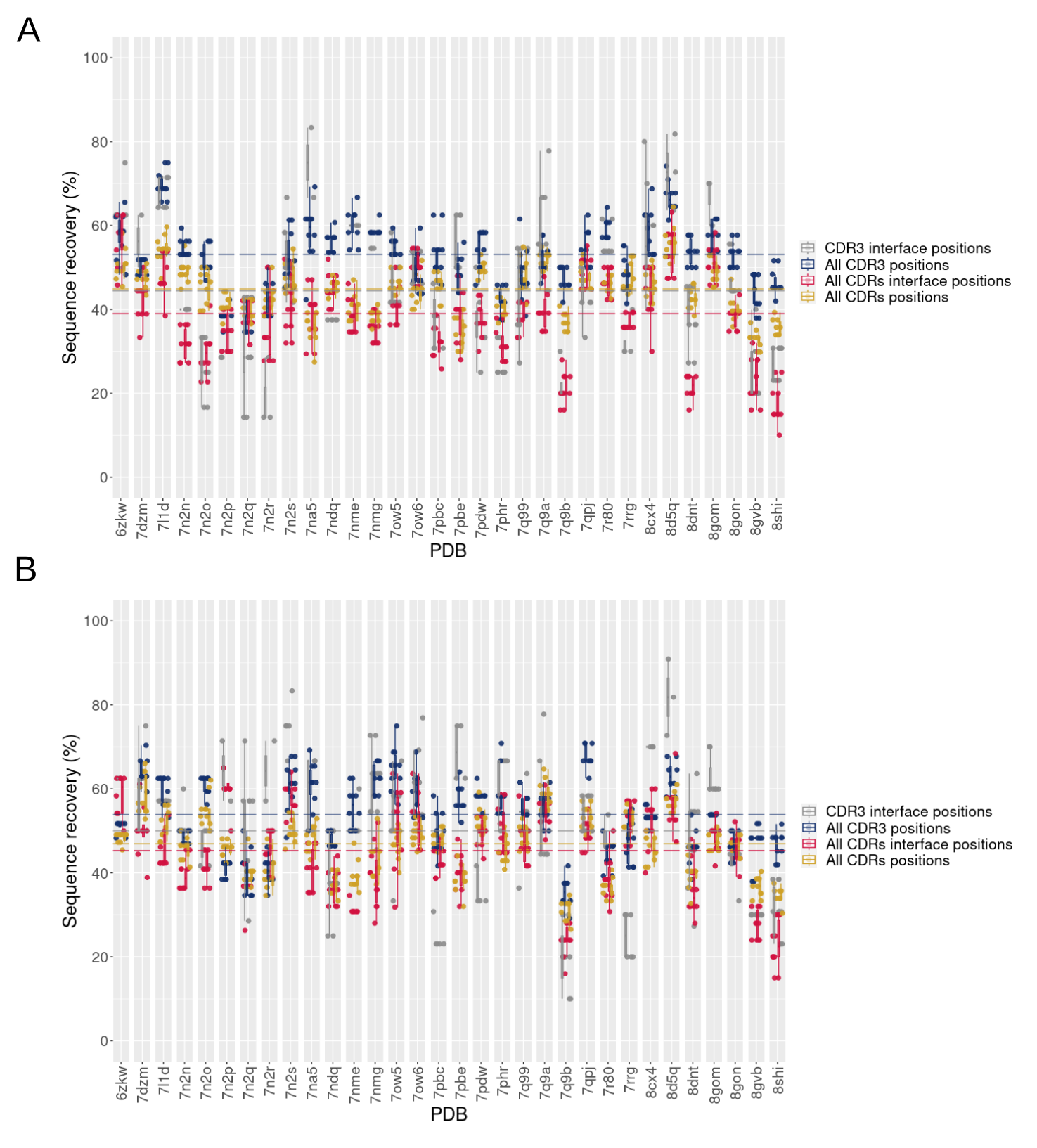

Supplement: S11 Fig — The box plots present the sequence recovery for each design strategy: design of only CDR3 (α and β chains) at the interface with the pMHC (in grey), design of all CDR3 (α and β chains) positions (in blue), design of CDR1, CDR2, and CDR3 positions (α and β chains) at the interface with the pMHC (in pink) and design of all CDR1, CDR2, and CDR3 positions (α and β chains) (in yellow). Each point corresponds to the sequence recovery of a designed sequence. Redundant sequences within each test case were excluded from the analysis. In (A), lines indicate the median computed over all designs: 44.4%, 53.1%, 39.0% and 44.9% for CDR3s interface positions, all CDR3 positions, all CDRs interface positions and all CDRs positions, respectively. In (B), lines indicate the median computed over all designs: 50.0%, 53.8%, 45.3% and 46.9% for CDR3s interface positions, all CDR3 positions, all CDR interface positions and all CDRs positions, respectively. (TIF) [file pcbi.1012489.s011.tif]

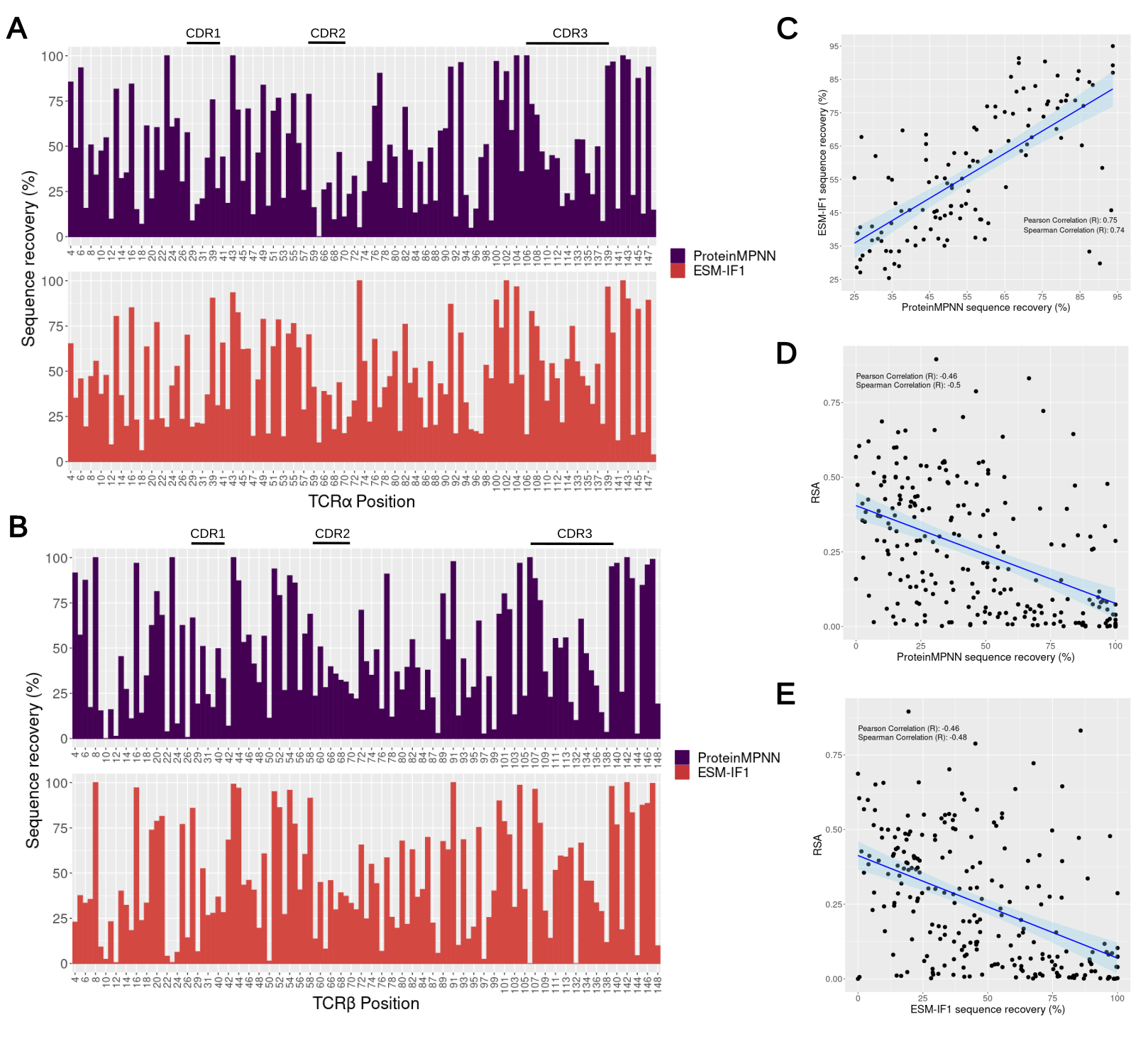

Supplement: S12 Fig — (A) Bar plots showing the averaged sequence recovery per position in the TCRα variable domain for ProteinMPNN (upper, purple) and ESM-IF1 (lower, red). This recovery considers all test cases and the designed sequences from these cases combined. (B) Identical to (A) but for the TCRβ variable domain. (C) A scatter plot with a linear trend line and a 95% confidence interval (light blue region) illustrates the correlation between sequence recoveries per position from ProteinMPNN and ESM-IF1 for the entire TCR variable domain design scenario. Each point represents a TCRαβ variable domain position, with correlation coefficients detailed on the plot. (D) Scatter plot with a linear trend line and a 95% confidence interval (light blue region) shows the correlation between sequence recovery per position from ProteinMPNN and the Relative Solvent Accessibility (RSA) for each position, with correlation coefficients detailed on the plot. (E) Similar to (D), this plot correlates RSA with ESM-IF1 sequence recovery per position. (TIF) [file pcbi.1012489.s012.tif]

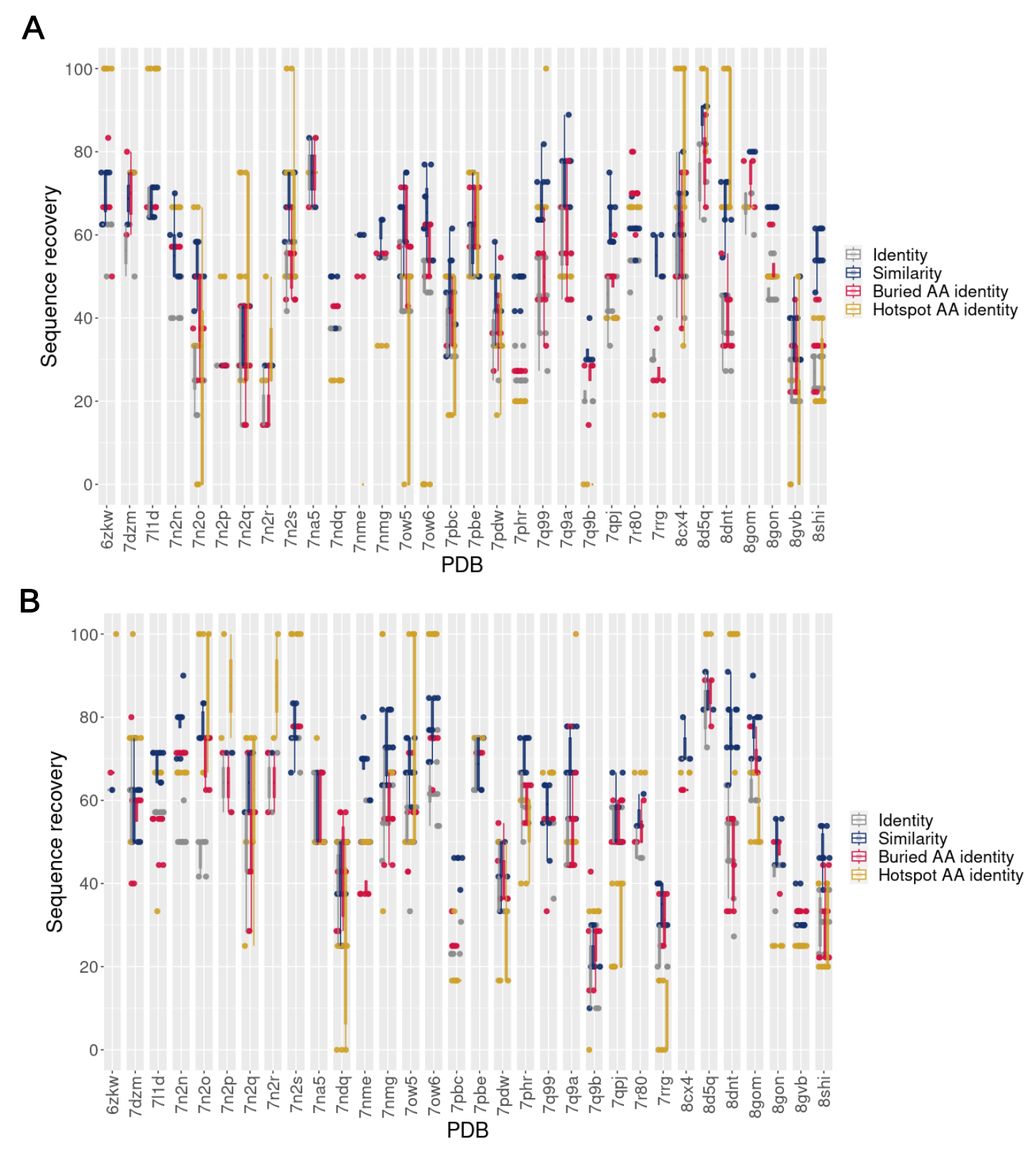

Supplement: S13 Fig — The sequence recovery (in %) is presented by box plots for each case designed by ProteinMPNN or ESM-IF1. Each point corresponds to a design sequence. (TIF) [file pcbi.1012489.s013.tif]

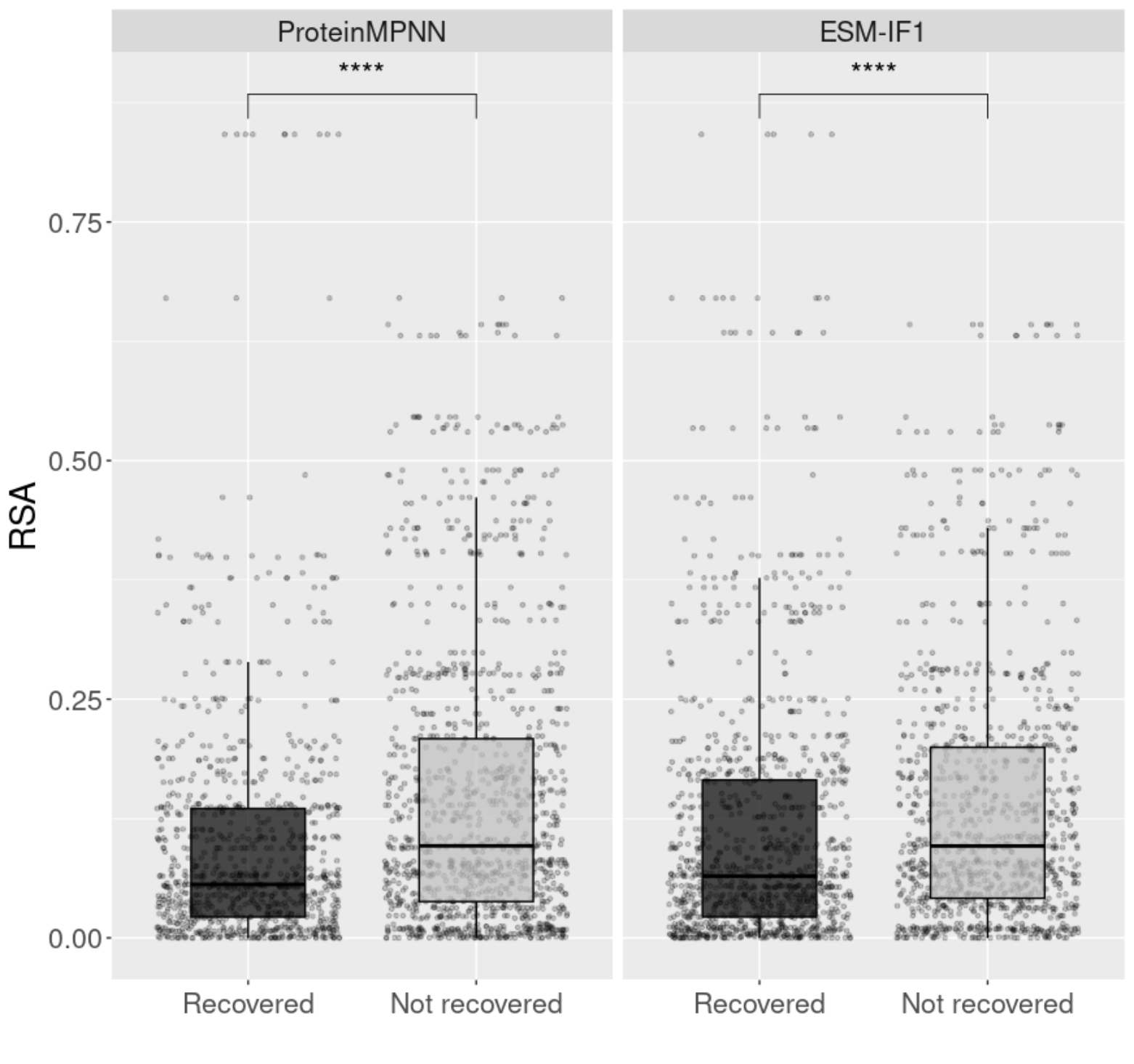

Supplement: S14 Fig — Each point represents a position that was either recovered or not during the design process with ProteinMPNN or ESM-IF1, along with the corresponding RSA. Statistical pairwise comparison assessed the significance between the identity (reference) and the other metrics. It was performed using the Mann-Whitney test with the R ggpubr package. Significance is indicated above each box plot (****, ** and * correspond to a p-value below 0.0001, 0.01 and 0.05, respectively, while ‘ns’ means no significance (p- value ≥ 0.05)). (TIF) [file pcbi.1012489.s014.tif]

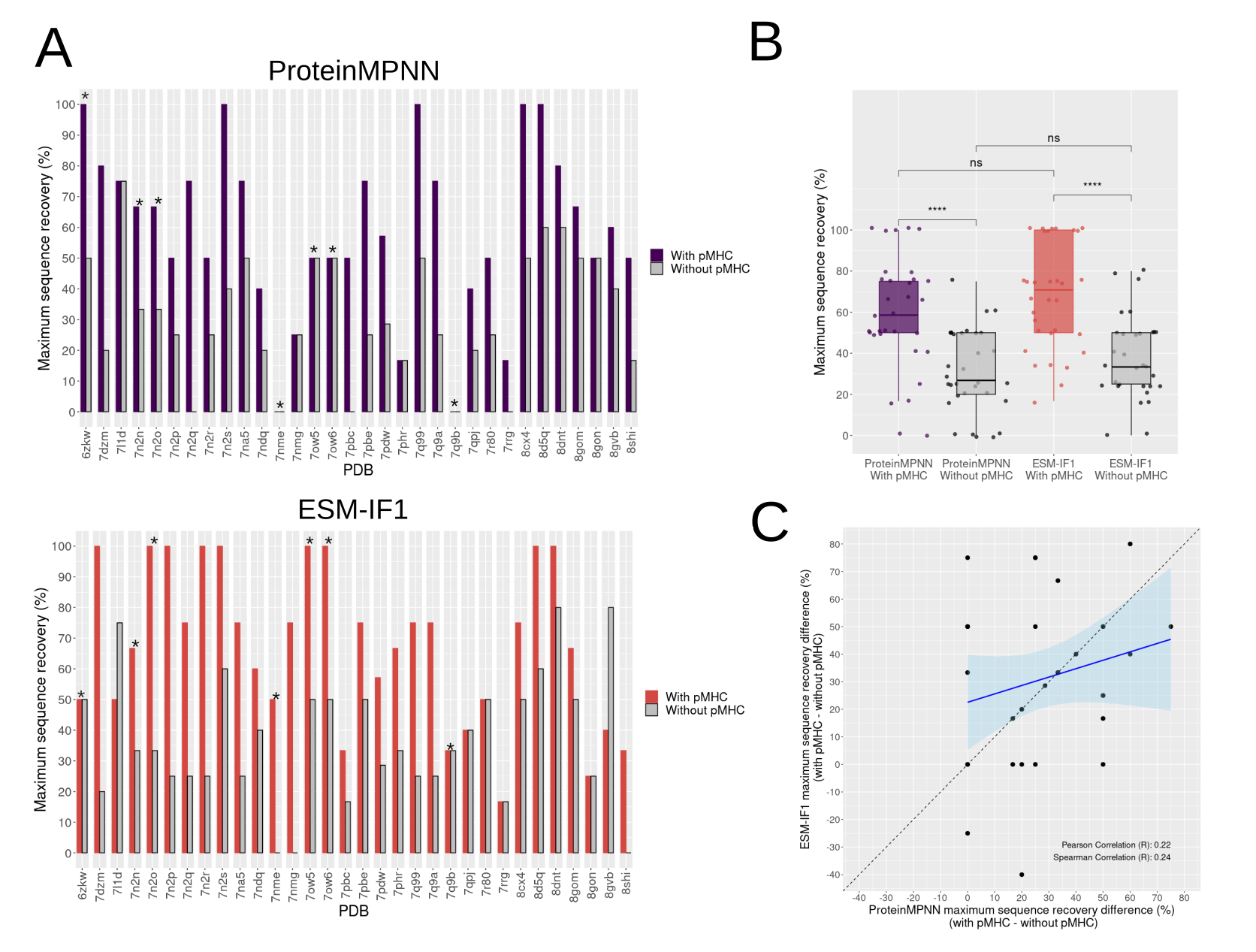

Supplement: S15 Fig — (A) Bar plot displaying the maximum sequence recovery for each test case, with designs generated by ProteinMPNN shown on the top and those by ESM-IF1 on the bottom. Colored bars (purple or red) represent the CDR3 interface designs considering the corresponding pMHC complex, while grey bars represent the interface design of unbound TCRs without the pMHC. Hotspot positions were predicted by computational alanine scanning experiments (see Methods) and an asterisk (*) indicates test cases that have fewer than four hotspot positions. (B) Same as (A), but grouping together the maximum sequence recovery values for ProteinMPNN with pMHC, ProteinMPNN without pMHC, ESM-IF1 with pMHC, and ESM-IF1 without pMHC. Statistical comparison between groups were performed using Mann-Whitney test with the R ggpubr package. Significance is indicated above each box plot (**** corresponds to a p-value below 0.0001, while ‘ns’ means no significance). (C) Scatter plot with a linear trend line and a 95% confidence interval (light blue region) illustrating the correlation between the difference in maximum sequence recovery upon pMHC removal (maximum sequence recovery with pMHC minus sequence recovery without pMHC) for ProteinMPNN and ESM-IF1. A dashed diagonal line is included to aid in visual comparison. The correlation coefficients are indicated in the plot. (TIF) [file pcbi.1012489.s015.tif]

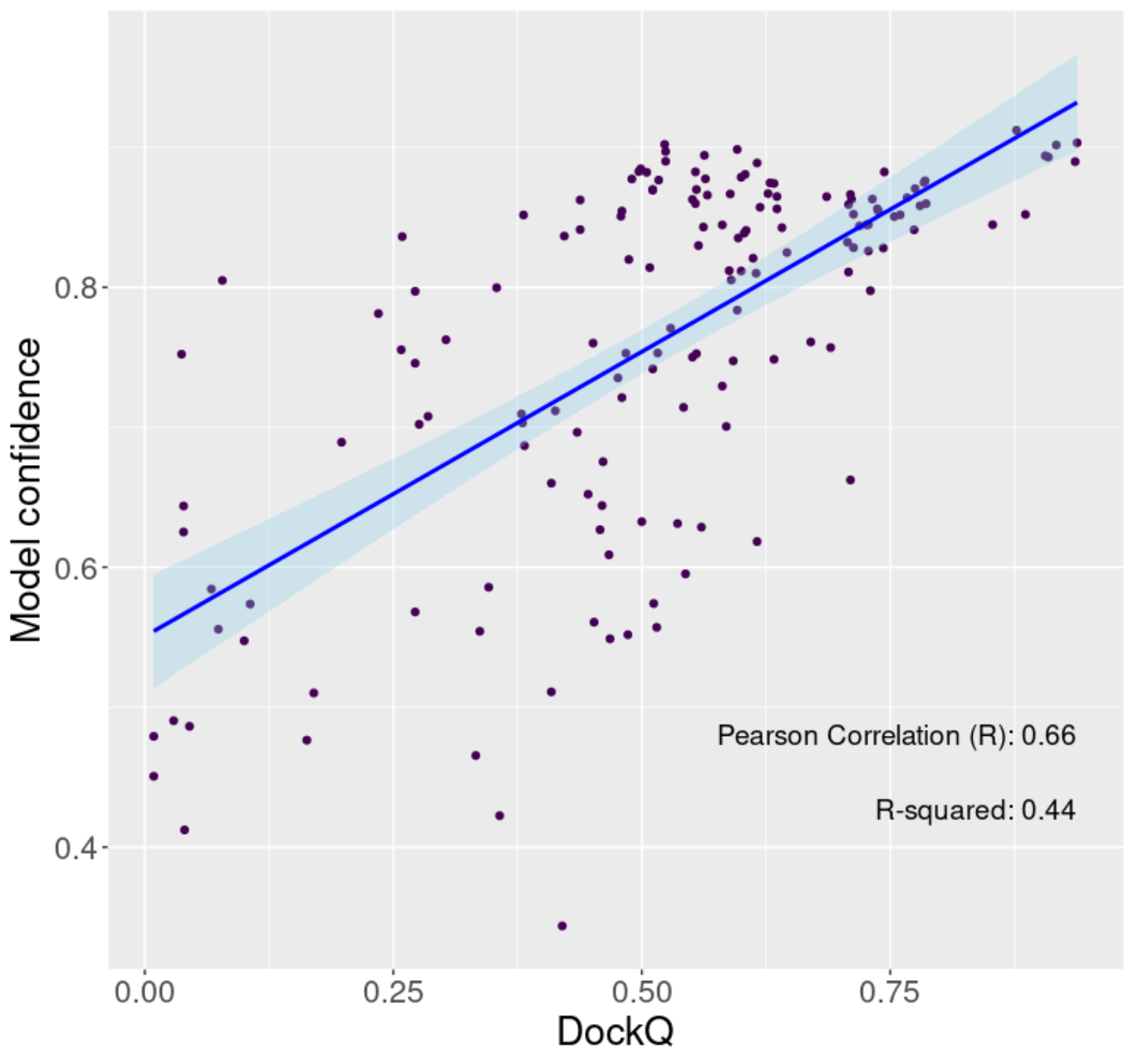

Supplement: S16 Fig — (TIF) [file pcbi.1012489.s016.tif]

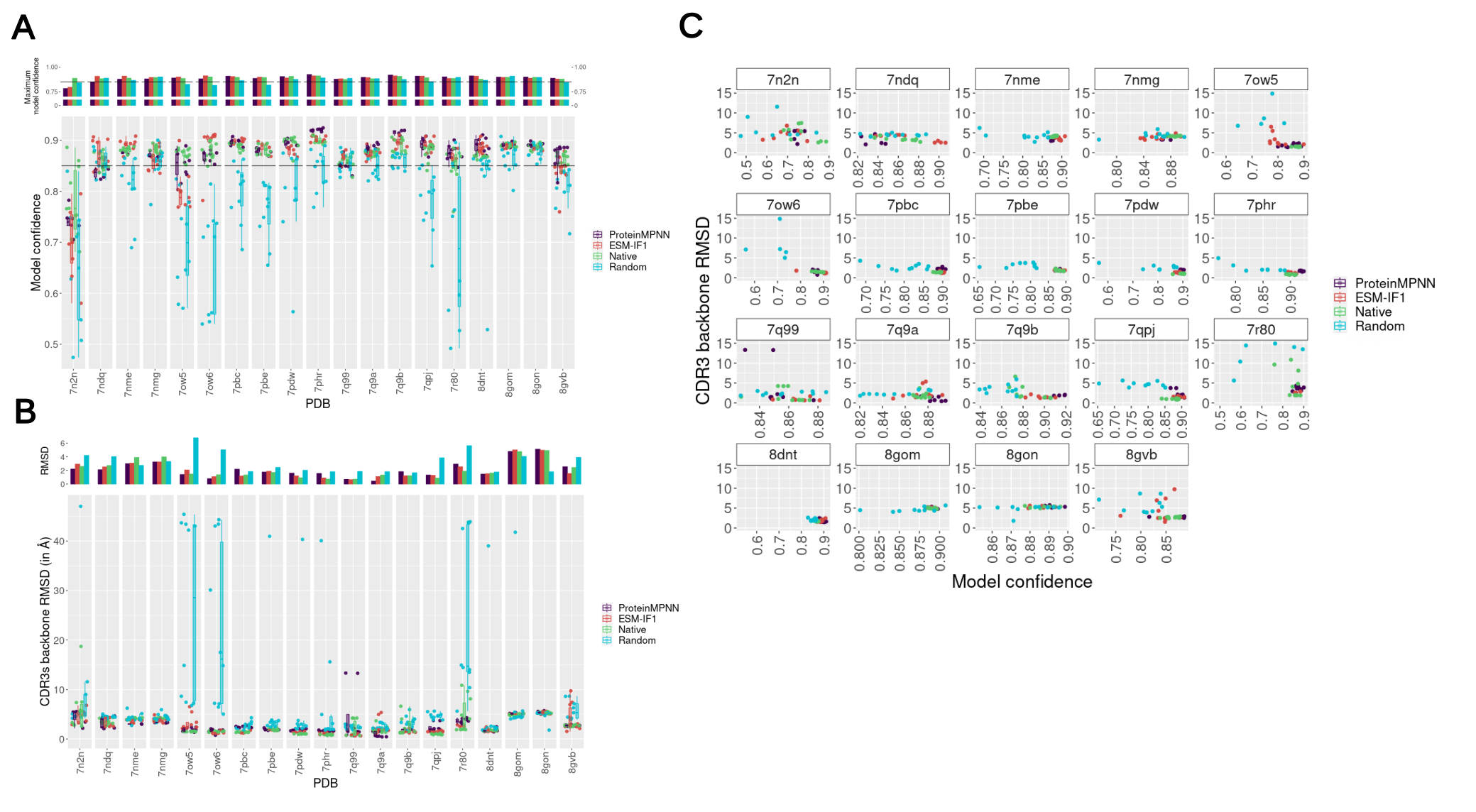

Supplement: S17 Fig — (A) Model confidence of ProteinMPNN (in purple), ESM-IF1 (in red) TCR designs, remodeled native structures (in green) and random dimissilar sequences (in cyan) for each test case. The secondary upper panel presents the maximum model confidence for each method and test case. (B) RMSD of CDR3 backbone atoms (both alpha and beta TCR chains) of designs and random in comparison to the corresponding native crystal structure that originated the designs. RMSDs were determined after structure superposition by the MHC. The secondary upper panel presents the minimum RMSD for each method and test case. (C) Scatter plot of the CDR3 backbone RMSD with the model confidence for ProteinMPNN and ESM-IF1 designs, random dissimilar sequences and native sequences. Since random sequences generate models with high deviation, for clarity only RMSD below 15 Å are presented. (TIF) [file pcbi.1012489.s017.tif]

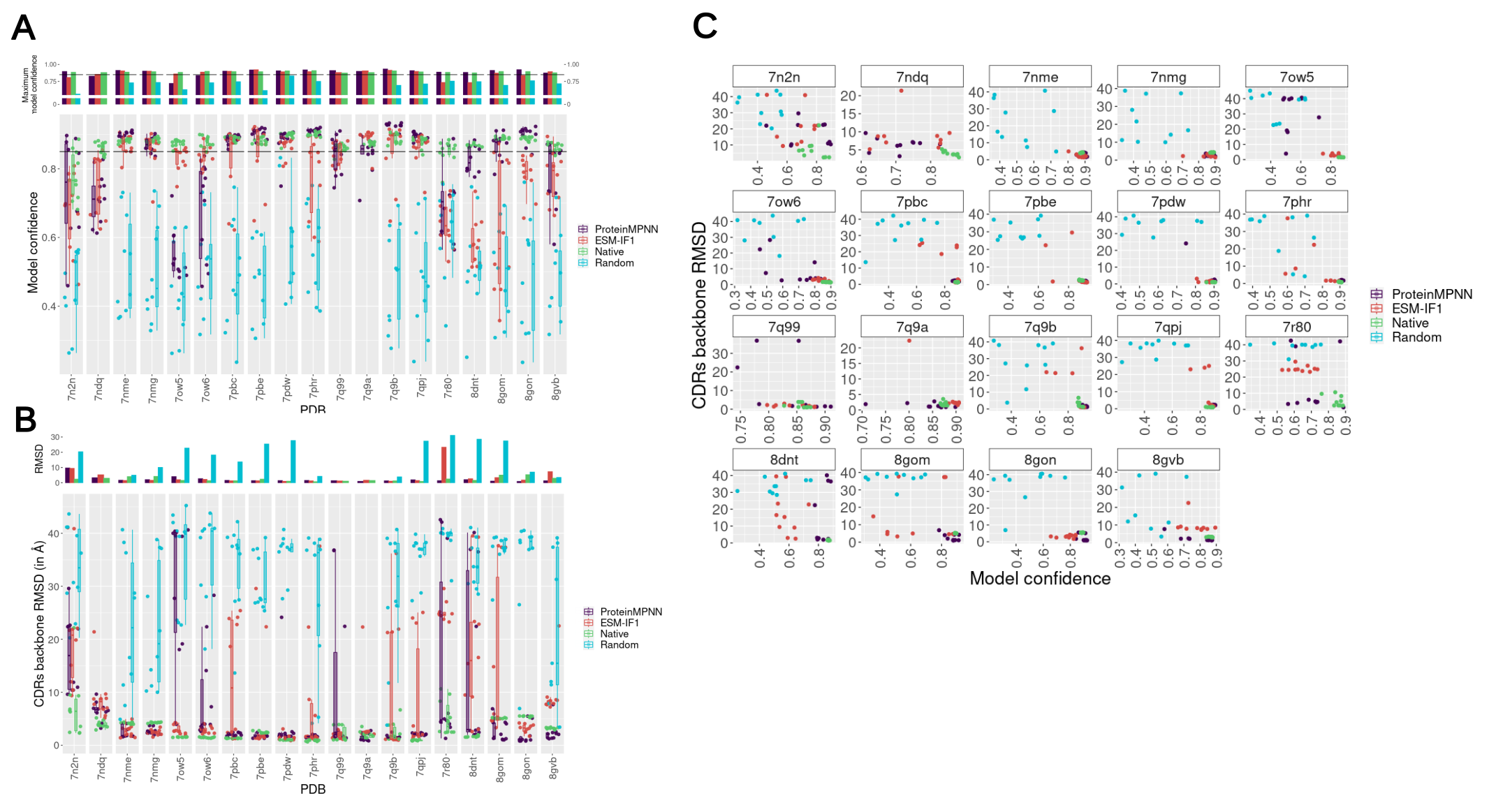

Supplement: S18 Fig — (A) Model confidence of ProteinMPNN (in purple), ESM-IF1 (in red) TCR designs, remodeled native structures (in green) and random dissimilar sequences (in cyan) for each test case. The secondary upper panel presents the maximum model confidence for each method and test case. Random dissimilar sequences from test cases 7ndq, 7q99 and 7q9a were not able to be modeled by TCRModel2. (B) RMSD of CDRs backbone atoms (both alpha and beta TCR chains) of designs and random in comparison to the corresponding native crystal structure that originated the designs. RMSDs were determined after structure superposition by the MHC. The secondary upper panel presents the minimum RMSD for each method and test case. (C) Scatter plot of the CDRs backbone RMSD with the model confidence for ProteinMPNN and ESM-IF1 designs, random dissimilar sequences and native sequences. (TIF) [file pcbi.1012489.s018.tif]

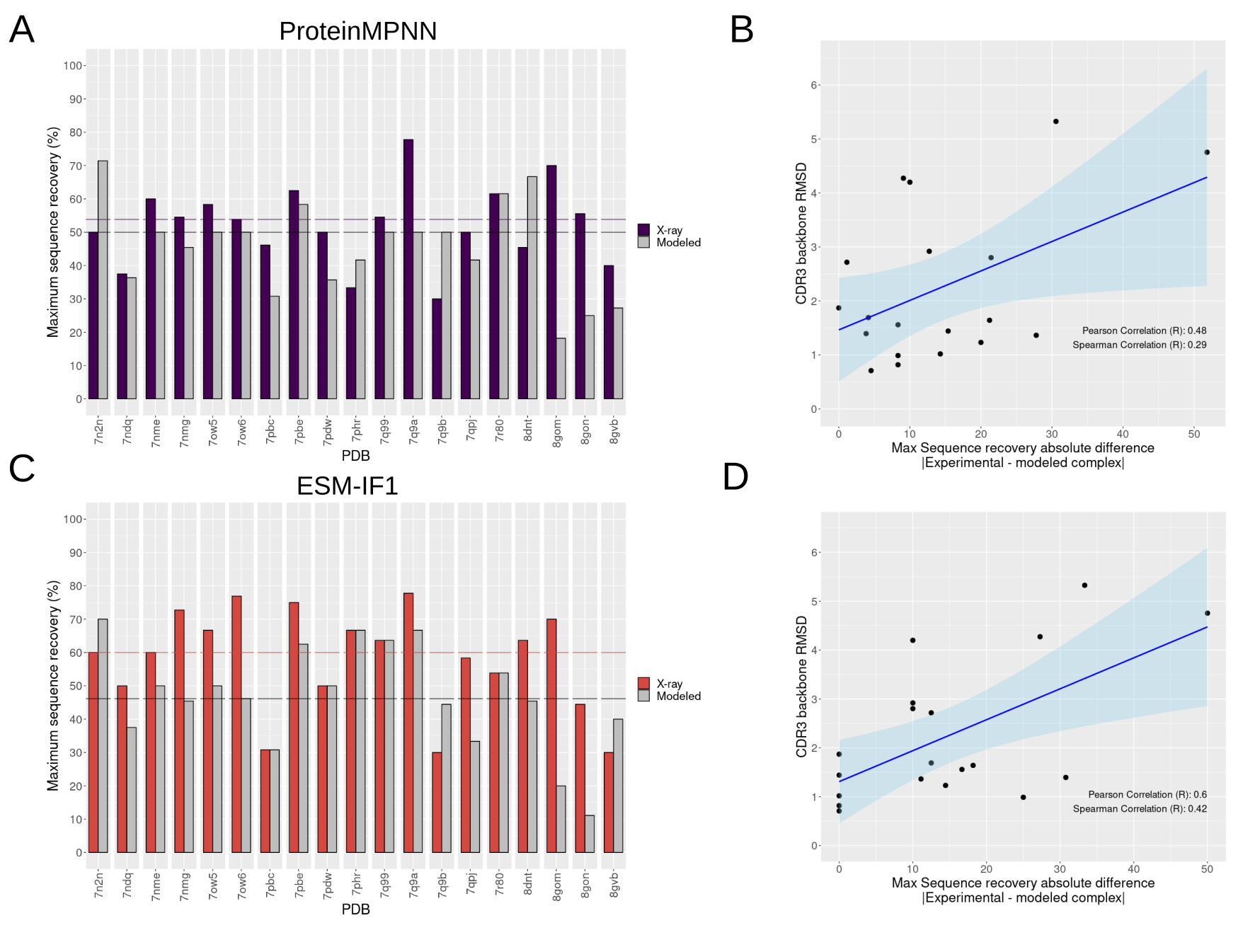

Supplement: S19 Fig — (A) Maximum sequence recovery (%) for each test case when designing sequences based on X-ray structures (purple bars) or structured modeled with TCRModel2 (grey bars). To obtain the maximum sequence recovery of modeled structures, we modeled the sequence of each test case 10 times with TCRModel2 and selected the top ranked model with the highest model confidence for each test case. Purple (for X-ray) and black (for modeled) dashed lines indicate the median of maximum sequence recovery of 53.8% and 50.0%, respectively, considering all test cases. (B) Scatter plot with a linear trend line and confidence interval of 0.95 (light blue region) presenting the correlation between the CDR3 backbone RMSD and the absolute difference in maximum sequence recovery from designs based on X-ray and modeled structures. The CDR3 backbone RMSD measures the deviation in the 3D positions of the CDR3 backbone between the X-ray and modeled structures. Correlation coefficients are detailed in the plot. (C) and (D) follow the same format as (A) and (B) but focus on ESM-IF1 designs. The median maximum sequence recovery values for ESM-IF1 are indicated by dashed lines, with 60.0% for X-ray and 46.1% for modeled structures. (TIF) [file pcbi.1012489.s019.tif]

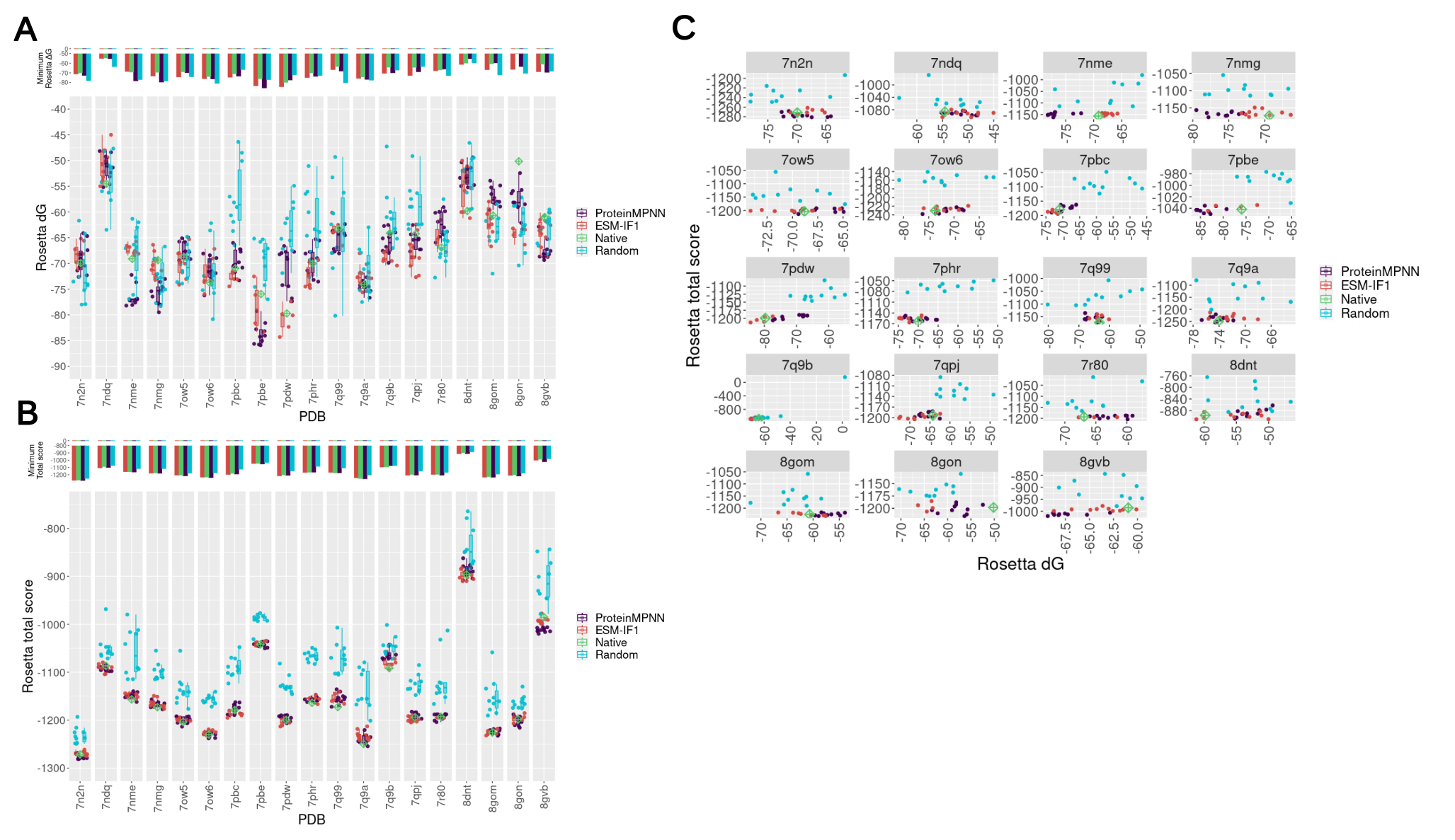

Supplement: S20 Fig — (A) Rosetta dG_separated term obtained from Rosetta InterfaceAnalyzer. Box plots represent dG_separated of the ESM-IF1 (in green) and ProteinMPNN (in purple) designs, dissimilar random generated TCRs (in cyan) and the red diamond corresponds to the dG_separated of the native structure The secondary upper panel presents the minimum Rosetta dG_separated for each method and test case. (B) Same as (A), but showing the Rosetta total_score term instead of dG_separated. The secondary upper panel presents the minimum Rosetta total_score for each method and test case. (C) Scatter plot presenting the relation between the dG_separated and total_score for native, designs and random sequences for all test cases. The lower the dG_separated and total_score scores, the higher the affinity and stabilization, respectively. (TIF) [file pcbi.1012489.s020.tif]

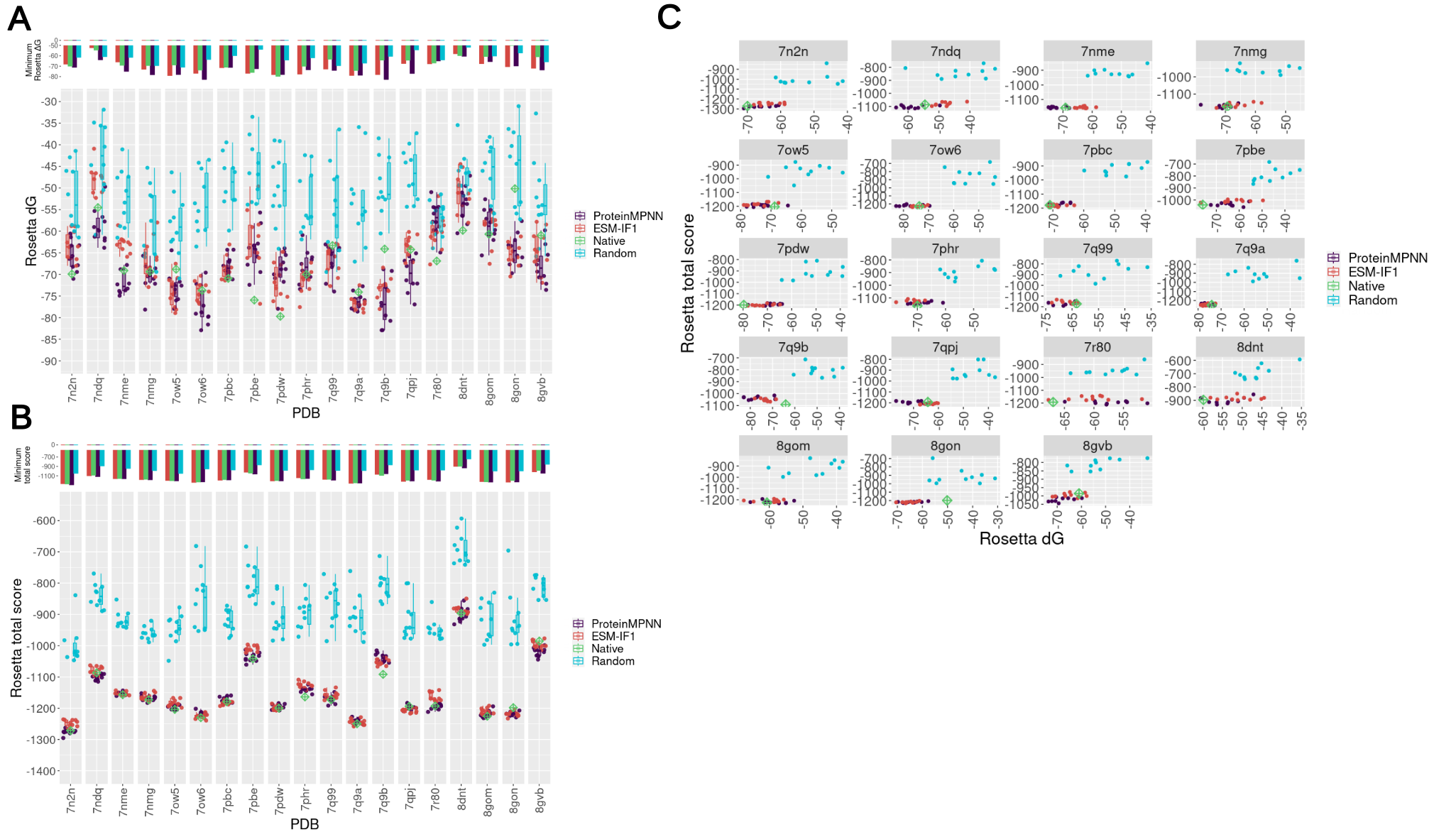

Supplement: S21 Fig — (A) Rosetta dG_separated term obtained from Rosetta InterfaceAnalyzer. Box plots represent dG_separated of the ESM-IF1 (in green) and ProteinMPNN (in purple) designs, dissimilar random generated TCRs (in cyan) and the red diamond corresponds to the dG_separated of the native structure. The secondary upper panel presents the minimum Rosetta dG_separated for each method and test case. (B) Same as (A), but showing the Rosetta total_score term instead of dG_separated. The secondary upper panel presents the minimum total_score for each method and test case. (C) Scatter plot presenting the relation between the dG_separated and total_score for native, designs and random sequences for all test cases. The lower the dG_separated and total_score scores, the higher the affinity and stabilization, respectively. (TIF) [file pcbi.1012489.s021.tif]

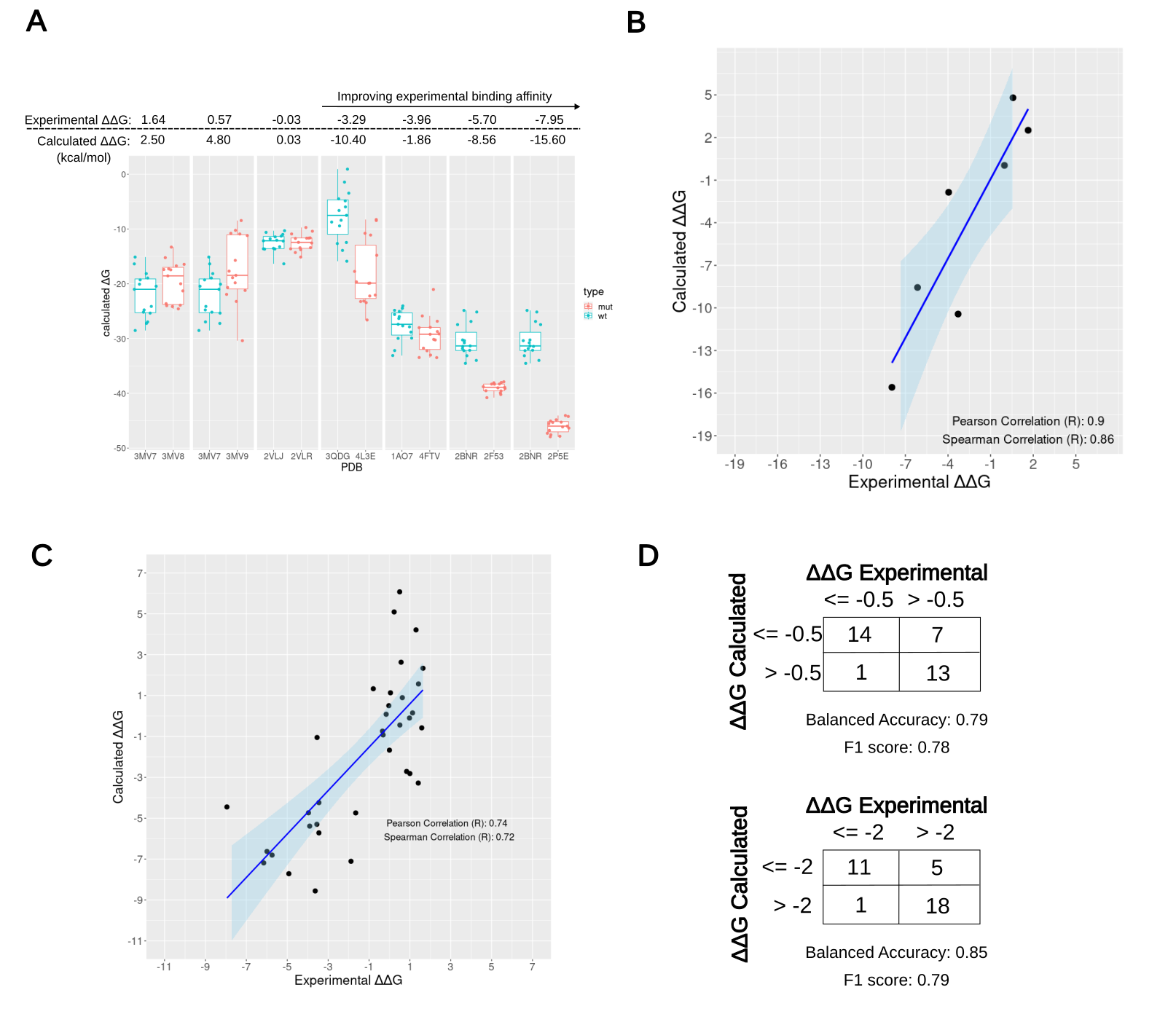

Supplement: S22 Fig — (A) Box plots of calculated ΔG (in kcal/mol) for the wild-type TCR complex (wt, in blue) or mutant complex (mut, in red) from MM/PBSA. Each point corresponds to a replica (15 in total) of a molecular dynamics simulation trajectory. The values of the experimental and calculated (ΔΔGmut − wt) (in kcal/mol) are shown above the box plot panel. (B) Scatter plot with a linear trend line and confidence interval of 0.95 (light blue region) presenting the correlation between the experimental and calculated ΔΔG considering the set of solved wild-type and mutant structures. Correlation coefficients are indicated in the plot. (C) Same as (B) but considering the set of modeled mutant structures. (D) Confusion matrix created from the scatter plot presented in (C) presenting the balanced accuracy and F1 score in the discrimination of ΔΔG greater than -0.5 kcal/mol (upper panel) or -2 kcal/mol (bottom panel). (TIF) [file pcbi.1012489.s022.tif]

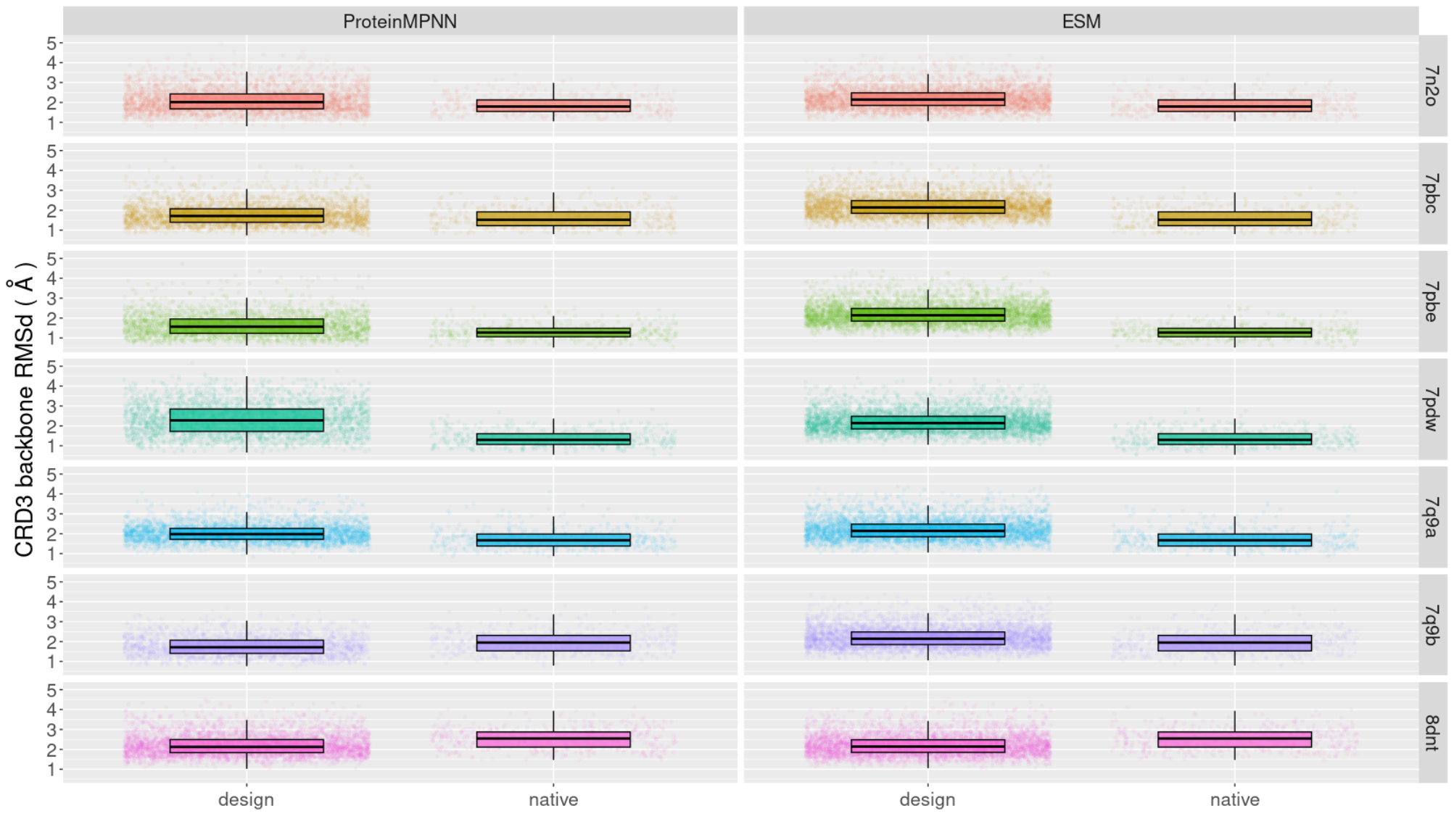

Supplement: S23 Fig — The RMSD values, depicted as box plots, were calculated subsequent to superposing the trajectory frames by the MHC of the corresponding reference crystal structure. For each test case, all replicas of all designs were combined to form a single RMSD distribution. (TIF) [file pcbi.1012489.s023.tif]

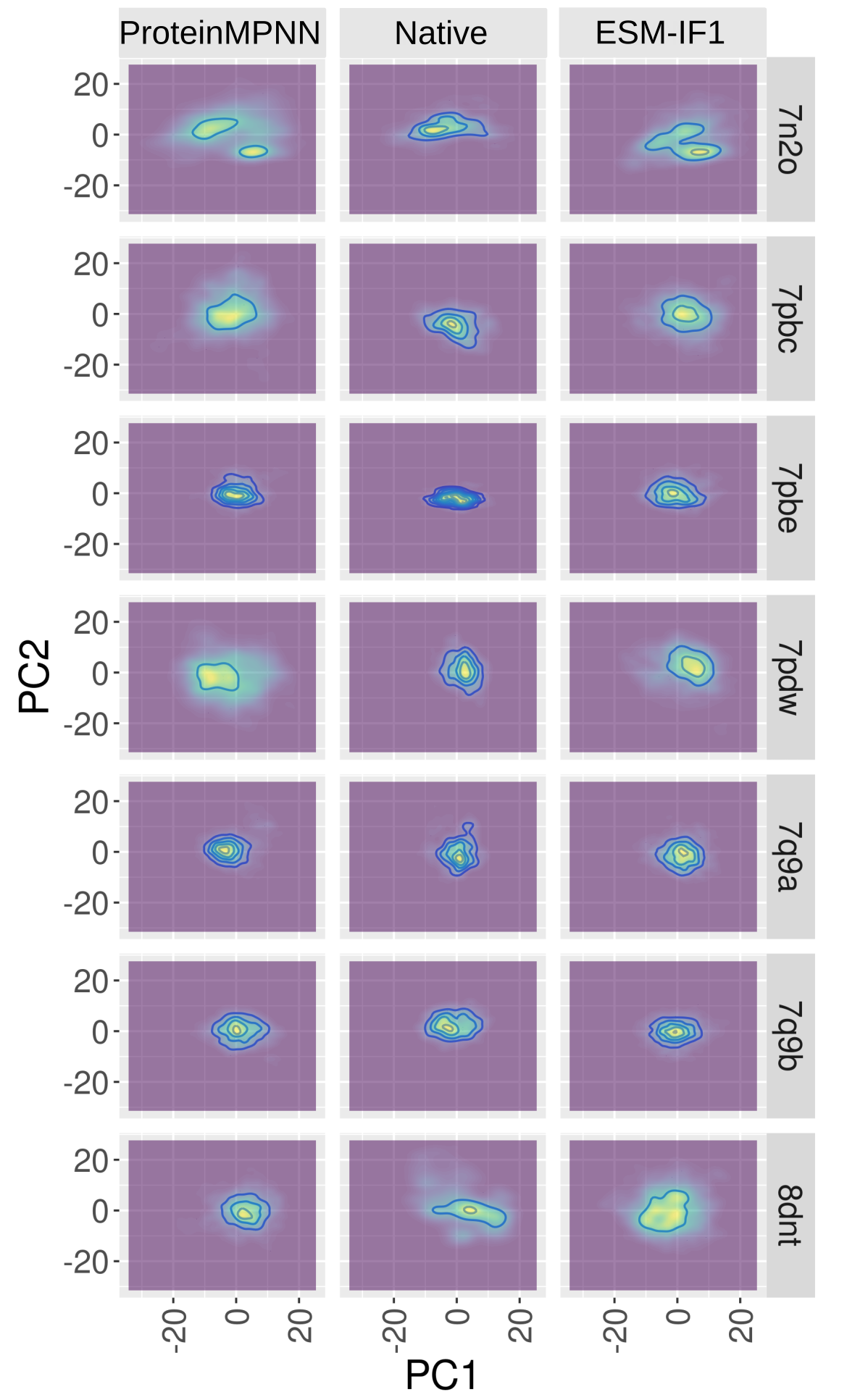

Supplement: S24 Fig — For the PCA analysis, only the CDR3 coordinates were considered (see Methods). The first two main components are represented as 2D density contours (bins of 50) colored by R viridis scale that ranges from dark purple to yellow, being yellow the regions of higher density. Plots were built using R ggplot package. (TIF) [file pcbi.1012489.s024.tif]

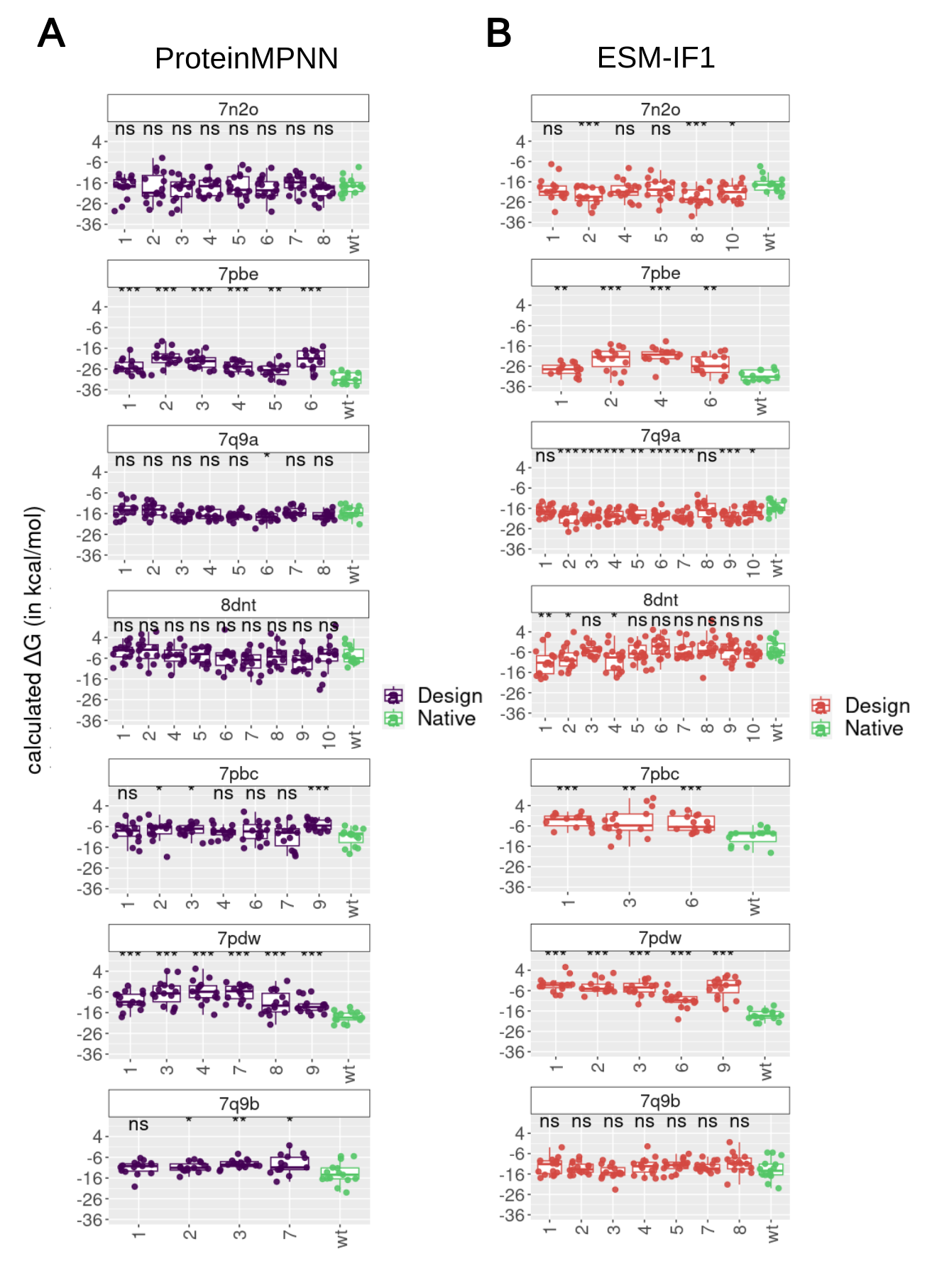

Supplement: S25 Fig — Each plot corresponds to a TCR:pMHC test case. The box plots present the ΔG (in kcal/mol) calculated for each of the 15 replicas of the TCR designs (ProteinMPNN in purple and ESM-IF1 in red) and the wild-type (wt) TCR (in green). The TCR designs are presented by IDs with a maximum of 10 designs. The lower number of designs are a consequence of redundant generated designs that were removed for the calculations. The statistical difference between each design and the corresponding wild-type was determined by Mann-Whitney test and the significance is indicated above each box plot (***, ** and * correspond to a p-value below 0.001, 0.01, and 0.05, respectively, while ‘ns’ means no significance). (TIF) [file pcbi.1012489.s025.tif]

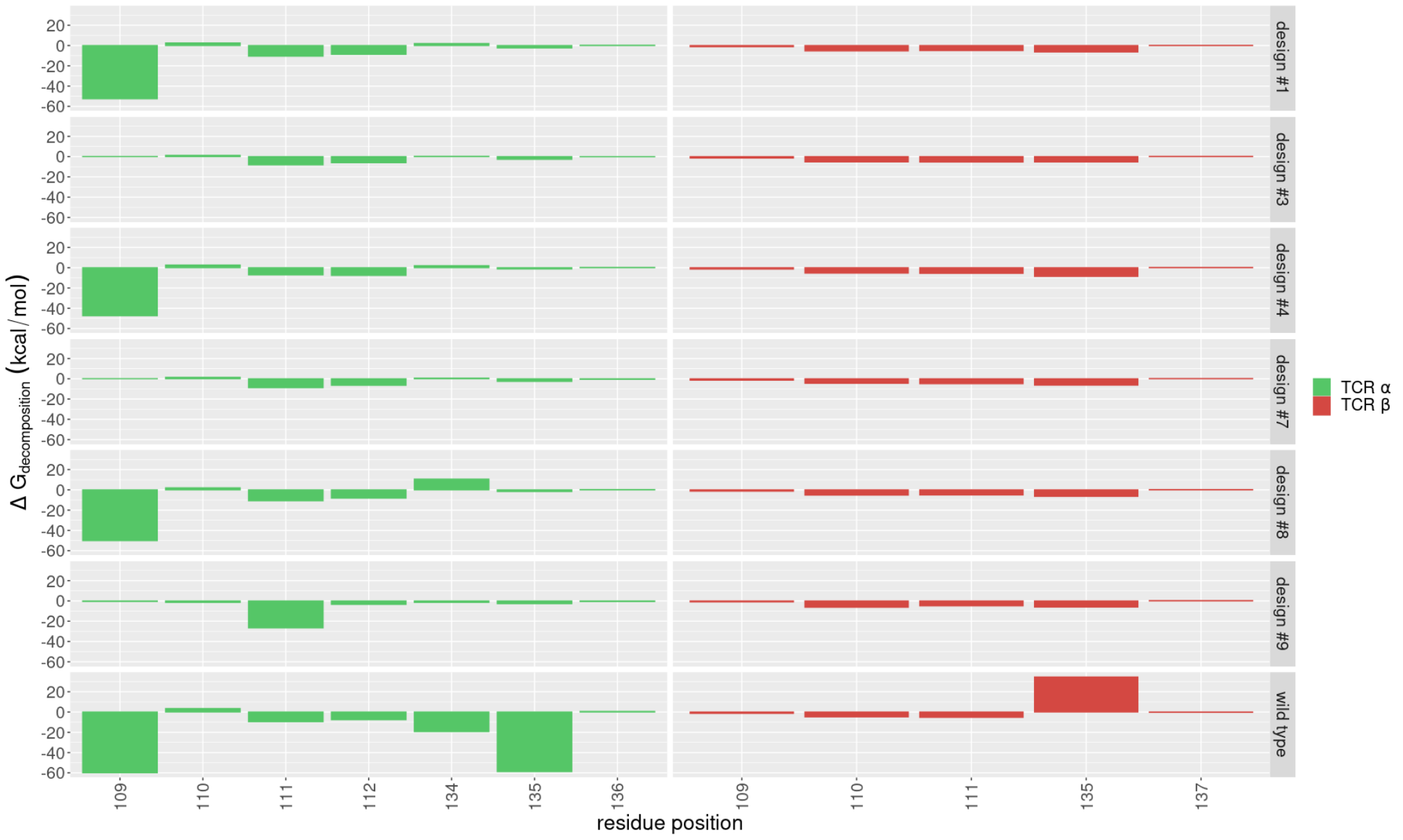

Supplement: S26 Fig — Residues from TCRα (green) and TCRβ (red) are shown. Low ΔG values indicate the residue contributed positively to the binding energy and high ΔG values indicate the residue contributed negatively to the binding energy. (TIF) [file pcbi.1012489.s026.tif]

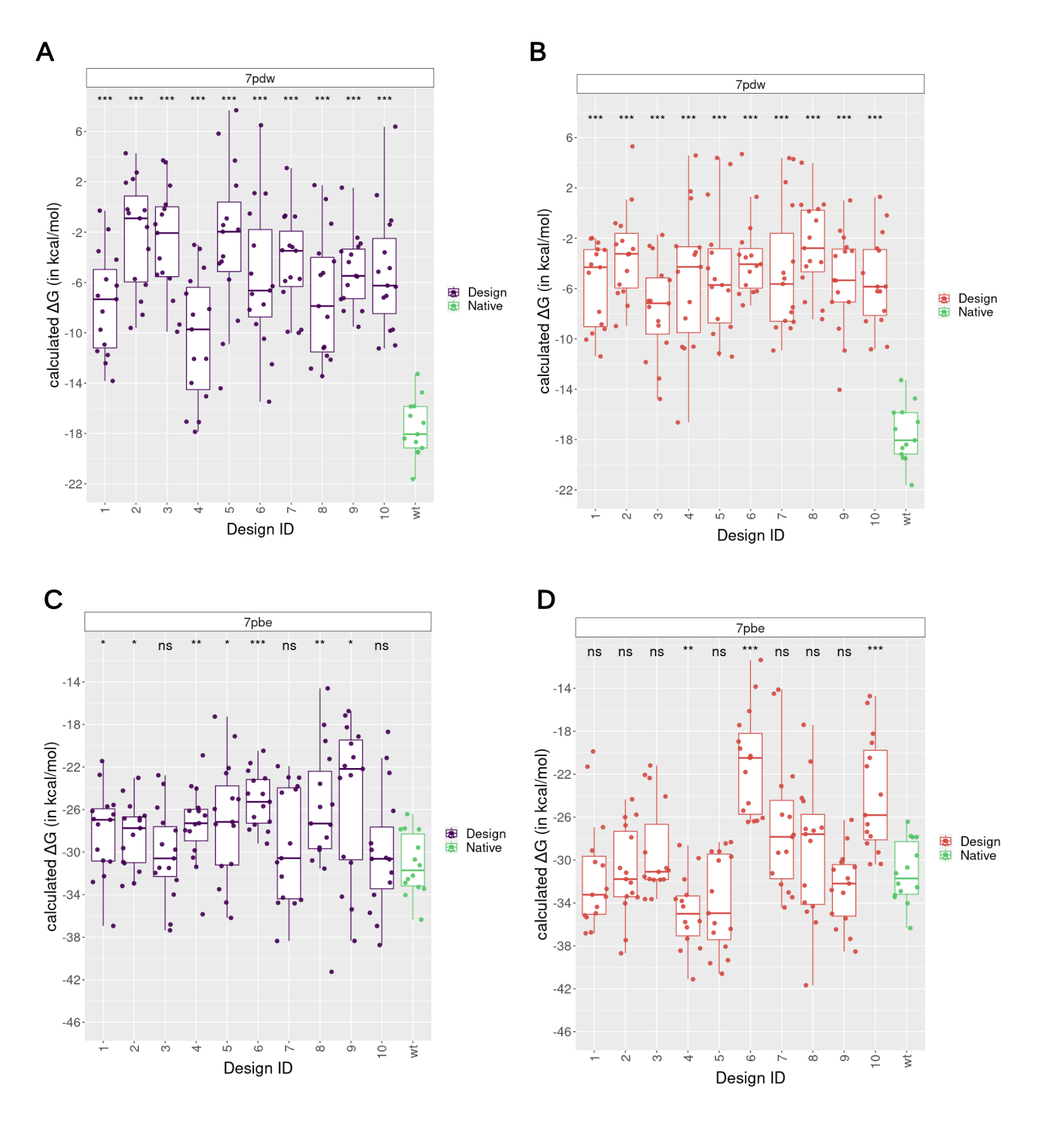

Supplement: S27 Fig — The box plots present the ΔG (in kcal/mol) calculated for each of the 15 replicas of the TCR designs (purple for ProteinMPNN and red for ESM-IF1) and the native TCR (in green). The TCR designs are presented by IDs with a maximum of 10 designs. The statistical difference between each design and the corresponding wild-type was determined by Mann-Whitney test and the significance is indicated above each box plot (***, ** and * correspond to a p-value below 0.001, 0.01, and 0.05, respectively, while ‘ns’ means no significance). (TIF) [file pcbi.1012489.s027.tif]

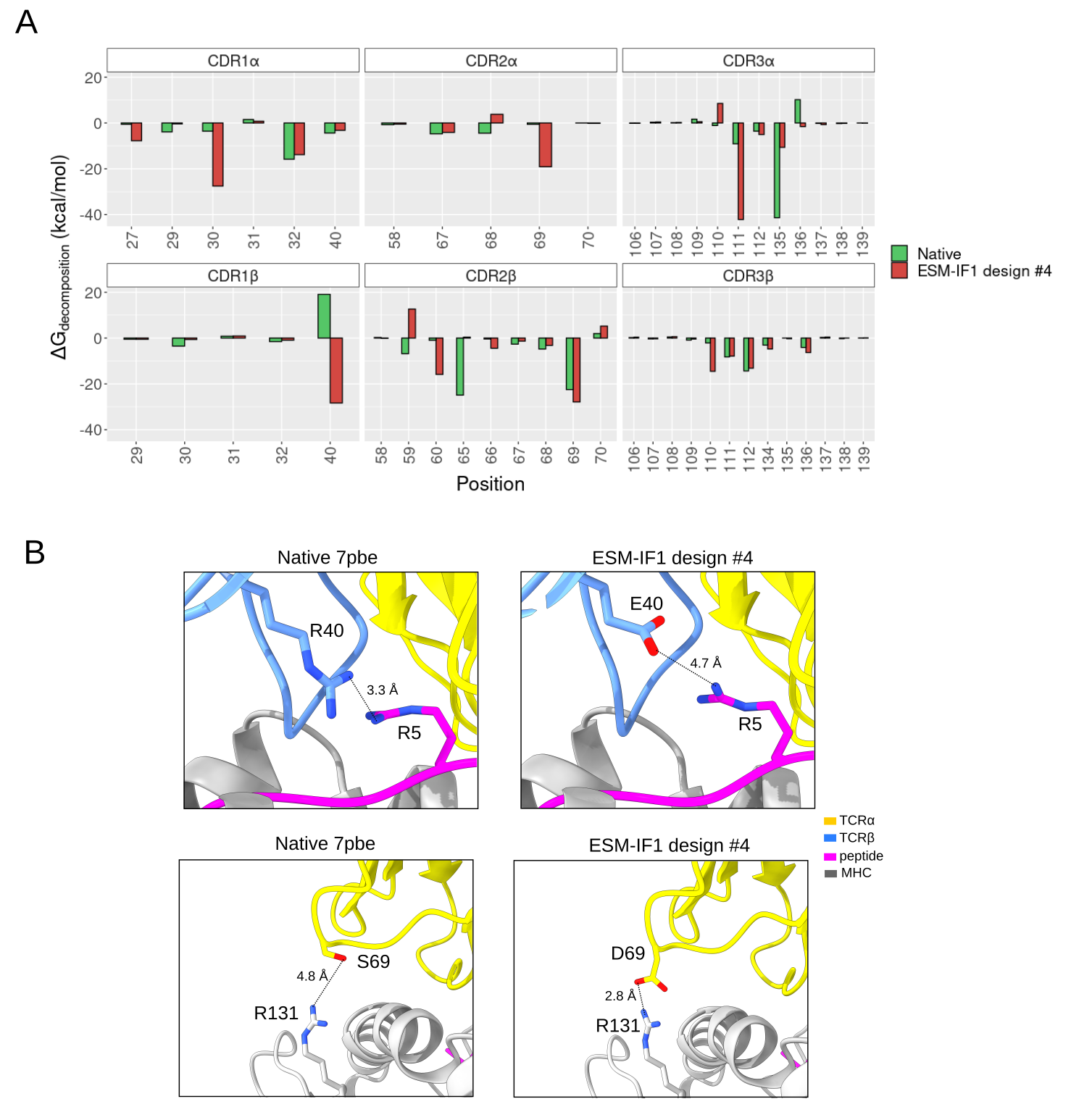

Supplement: S28 Fig — (A) Free energy decomposition (in kcal/mol) per CDR position for the native 7pbe complex (green) and the ESM-IF1 design #4 (red). Panels are split by CDR regions. Low ΔG values indicate residues that contributed positively to the binding energy, while high ΔG values indicate residues that contributed negatively. (B) Atomic contacts observed in the native 7pbe complex (first column panels) and in the ESM-IF1 design #4 (second column panels). The TCRα chain is shown in yellow, the TCRβ chain in blue, the peptide in magenta, and the MHC in grey. Residues involved in the highlighted contacts are represented as sticks, with atomic distances indicated. The atomic structures presented were obtained through energy minimization. (TIF) [file pcbi.1012489.s028.tif]
