# Supplementary material for: Exploring the potential of structure-based deep learning approaches for T cell receptor design
Source: PLoS Comput Biol. 2024 Sep 30;20(9):e1012489. doi: 10.1371/journal.pcbi.1012489 (PMC11466415; doi:10.1371/journal.pcbi.1012489)
Supplement: S1 Appendix — (PDF) [file pcbi.1012489.s029.pdf]

S1 Appendix. Rosetta resfile example.

```
NATAA
start
110 D ALLAAxc
111 D ALLAAxc
112 D ALLAAxc
134 D ALLAAxc
135 D ALLAAxc
113 E ALLAAxc
114 E ALLAAxc
133 E ALLAAxc
```
