# Supplementary material for: Exploring the potential of structure-based deep learning approaches for T cell receptor design
Source: PLoS Comput Biol. 2024 Sep 30;20(9):e1012489. doi: 10.1371/journal.pcbi.1012489 (PMC11466415; doi:10.1371/journal.pcbi.1012489)
Supplement: S2 Appendix — (PDF) [file pcbi.1012489.s030.pdf]

**S2 Appendix. Rosetta design protocol.**

```
<ROSETTASCRIPTS>
  <SCOREFXNS>
    <ScoreFunction name="r15" weights="ref2015.wts" />
  </SCOREFXNS>
  <RESIDUE_SELECTORS>
    </RESIDUE_SELECTORS>
  <TASKOPERATIONS>
    <ReadResfile name="rrf" filename="rrf.resfile" />
  </TASKOPERATIONS>
  <SIMPLE_METRICS>
    </SIMPLE_METRICS>
  <FILTERS>
    </FILTERS>
  <MOVERS>
    <InterfaceAnalyzerMover name="int_analyzer" scorefxn="REF2015" interface="DE_CA" />
    <FastDesign name="fastdesign" disable_design="false" task_operations="rrf" relaxscript="InterfaceDesign2019" repeats="3" scorefxn="r15" />
  </MOVERS>
  <PROTOCOLS>
    <Add mover="fastdesign" />
    <Add mover="int_analyzer" />
  </PROTOCOLS>
  <OUTPUT />
</ROSETTASCRIPTS>
```
