# Supplementary material for: Exploring the potential of structure-based deep learning approaches for T cell receptor design
Source: PLoS Comput Biol. 2024 Sep 30;20(9):e1012489. doi: 10.1371/journal.pcbi.1012489 (PMC11466415; doi:10.1371/journal.pcbi.1012489)
Supplement: S3 Appendix — (PDF) [file pcbi.1012489.s031.pdf]

**S3 Appendix. Rosetta2.3 command line for alanine scanning.**

```
$ rosetta++/bin/rosetta.release aa . input.pdb -interface -intout_by_pdb
↪ -ignore_unrecognized_res -safety_check -skip_missing_residues -mutlist mut_list_file
↪ -min_interface -int_chi -extrachi_cutoff 1 -ex1 -ex2 -ex3 -constant_seed -jran 14
↪ -yap -s input.pdb -paths paths.txt
```
