# Supplementary material for: Exploring the potential of structure-based deep learning approaches for T cell receptor design
Source: PLoS Comput Biol. 2024 Sep 30;20(9):e1012489. doi: 10.1371/journal.pcbi.1012489 (PMC11466415; doi:10.1371/journal.pcbi.1012489)
Supplement: S4 Appendix — (PDF) [file pcbi.1012489.s032.pdf]

S4 Appendix. Example of mutation list file for Rosetta2.3 alanine scanning protocol.

```
START
13
1
MUTATIONS 109  D  R  A
1
MUTATIONS 110  D  G  A
1
MUTATIONS 111  D  T  A
1
MUTATIONS 112  D  G  A
1
MUTATIONS 134  D  R  A
1
MUTATIONS 135  D  R  A
1
MUTATIONS 136  D  A  A
1
MUTATIONS 109  E  S  A
1
MUTATIONS 110  E  F  A
1
MUTATIONS 111  E  T  A
1
MUTATIONS 135  E  D  A
1
MUTATIONS 136  E  T  A
1
MUTATIONS 137  E  Q  A
```
