# Supplementary material for: Exploring the potential of structure-based deep learning approaches for T cell receptor design
Source: PLoS Comput Biol. 2024 Sep 30;20(9):e1012489. doi: 10.1371/journal.pcbi.1012489 (PMC11466415; doi:10.1371/journal.pcbi.1012489)
Supplement: S5 Appendix — (PDF) [file pcbi.1012489.s033.pdf]

**S5 Appendix. Rosetta interface analyzer protocol.**

```
<ROSETTASCRIPTS>
<SCOREFXNS>
    <ScoreFunction name="ref" weights="ref2015.wts">
    <Reweight scoretype="coordinate_constraint" weight="0.5" />
    </ScoreFunction>
</SCOREFXNS>
<RESIDUE_SELECTORS>
    <Index name="sel_res1" resnums="111D" />
    <Index name="sel_res2" resnums="112D" />
    <Index name="sel_res3" resnums="113D" />
    <Index name="sel_res4" resnums="114D" />
    <Index name="sel_res5" resnums="135D" />
    <Index name="sel_res6" resnums="112E" />
    <Index name="sel_res7" resnums="113E" />
    <Index name="sel_res8" resnums="133E" />
    <Index name="sel_res9" resnums="134E" />
    <Index name="sel_res10" resnums="135E" />
</RESIDUE_SELECTORS>
<TASKOPERATIONS>
    </TASKOPERATIONS>
<SIMPLE_METRICS>
    </SIMPLE_METRICS>
<FILTERS>
    </FILTERS>
<MOVERS>
    <MutateResidue mutate_self="False" name="mut1" new_res="LEU"
    ↪  preserve_atom_coords="False" residue_selector="sel_res1" />
    <MutateResidue mutate_self="False" name="mut2" new_res="THR"
    ↪  preserve_atom_coords="False" residue_selector="sel_res2" />
    <MutateResidue mutate_self="False" name="mut3" new_res="PHE"
    ↪  preserve_atom_coords="False" residue_selector="sel_res3" />
    <MutateResidue mutate_self="False" name="mut4" new_res="ILE"
    ↪  preserve_atom_coords="False" residue_selector="sel_res4" />
    <MutateResidue mutate_self="False" name="mut5" new_res="PRO"
    ↪  preserve_atom_coords="False" residue_selector="sel_res5" />
    <MutateResidue mutate_self="False" name="mut6" new_res="ARG"
    ↪  preserve_atom_coords="False" residue_selector="sel_res6" />
    <MutateResidue mutate_self="False" name="mut7" new_res="ASN"
    ↪  preserve_atom_coords="False" residue_selector="sel_res7" />
    <MutateResidue mutate_self="False" name="mut8" new_res="VAL"
    ↪  preserve_atom_coords="False" residue_selector="sel_res8" />
    <MutateResidue mutate_self="False" name="mut9" new_res="LEU"
    ↪  preserve_atom_coords="False" residue_selector="sel_res9" />
    <MutateResidue mutate_self="False" name="mut10" new_res="VAL"
    ↪  preserve_atom_coords="False" residue_selector="sel_res10" />
    <AtomCoordinateCstMover bounded="false" coord_dev="0.5"
    ↪  name="constrain_relax_to_start_coords" sidechain="false" />
    <VirtualRoot name="vroot" removable="true" remove="false" />
    <FastRelax delete_virtual_residues_after_FastRelax="true" name="FastRelax"
    ↪  ramp_down_constraints="false" scorefxn="ref" />
    <InterfaceAnalyzerMover interface="DE_AC" name="int_analyzer" scorefxn="REF2015" />
</MOVERS>
```

```
<PROTOCOLS>
    <Add mover="mut1" />
    <Add mover="mut2" />
    <Add mover="mut3" />
    <Add mover="mut4" />
    <Add mover="mut5" />
    <Add mover="mut6" />
    <Add mover="mut7" />
    <Add mover="mut8" />
    <Add mover="mut9" />
    <Add mover="mut10" />
    <Add mover="vroot" />
    <Add mover="constrain_relax_to_start_coords" />
    <Add mover="FastRelax" />
    <Add mover="int_analyzer" />
</PROTOCOLS>
<OUTPUT />
</ROSETTASCRIPTS>
```
