# Supplementary material for: Exploring the potential of structure-based deep learning approaches for T cell receptor design
Source: PLoS Comput Biol. 2024 Sep 30;20(9):e1012489. doi: 10.1371/journal.pcbi.1012489 (PMC11466415; doi:10.1371/journal.pcbi.1012489)
Supplement: S1 Table — (PDF) [file pcbi.1012489.s034.pdf]

S1 Table. List of PDB IDs of MHC-I and MHC-II TCR:pMHC complexes used for comparing design methods.

MHC Class I						
**PDB**	**CDR3α**	**CDR3β**	**Release date**	**Resolution**	**CDR3α length**	**CDR3β length**
6ZKW	CAVTNQAGTALIF	CASSYSIRGSRGEQFF	01/26/22	2.26	13	16
7DZM	CIVRGLNNAGNMLTF	CASSLGIDAIYF	01/26/22	2.25	15	12
7L1D	CAGNTGTASKLTF	CASSGLAGGPVSGANVLTF	03/23/22	3.11	13	19
7N2N	CAVLSPVQETSGSRLTF	CASSVGLFSTDTQYF	12/07/22	2.6	17	15
7N2O	CAVLSPVQETSGSRLTF	CASSVGLFSTDTQYF	12/07/22	2.3	17	15
7N2P	CAVSNFNKFYF	CASSVATYSTDTQYF	12/07/22	2.5	11	15
7N2Q	CAVSNFNKFYF	CASSVATYSTDTQYF	12/07/22	2.7	11	15
7N2R	CAVSNFNKFYF	CASSVATYSTDTQYF	12/07/22	2.28	11	15
7N2S	CAVSLGTGAGSYQLTF	CASSVGLYSTDTQYF	12/07/22	2.37	16	15
7NA5	CAVSNYNVLYF	CASSQEPGGYAEQFF	06/22/22	2.5	11	15
7NDQ	CLVGSSFNQGGKLIF	CASSLGREYGYTF	01/26/22	2.55	15	13
7NME	CAEPSGNTGKLIF	CASSLHHEQYF	09/07/22	2.2	13	11
7NMG	CAEPSGNTGKLIF	CASSLHHEQYF	09/07/22	2.48	13	11
7OW5	CAMSVPSGDGSYQFTF	CASKVGPGQHNSPLHF	07/20/22	2.58	16	16
7OW6	CAMSVPSGDGSYQFTF	CASKVGPGQHNSPLHF	07/20/22	2.64	16	16
7PBC	CAVRGTGRRALTF	CASSFTDTQYF	08/03/22	2.04	13	11
7PBE	CVVNINTDKLIF	CASSSANSGELFF	04/27/22	3	12	13
7PDW	CAVRGTGRRALTF	CASSFTDTQYF	08/03/22	1.82	13	11
7PHR	CATDGSTPMQF	CASSWGAPYEQYF	08/31/22	3.08	11	13
7Q99	CAVNVAGKSTF	CAWSETGLGTGELFF	02/22/23	2.55	11	15
7Q9A	CAVNVAGKSTF	CAWSETGLGTGELFF	02/22/23	2.1	11	15
7Q9B	CAVQKLVF	CASSYSFTEATYEQYF	02/22/23	3.24	8	16
7QPJ	CAVRGTGRRALTF	CASSFATEAFF	08/03/22	1.54	13	11
7R80	CAQLNQAGTALIF	CASSYGTGINYGYTF	06/29/22	2.9	13	15
7RRG	CLVGGAYTGGFKTIF	CASSLVAETYEQYF	03/23/22	2.12	15	14
8CX4	CAVNSPGSGAGSYQLTF	CASSVGTYSTDTQYF	12/07/22	2.2	17	15
8D5Q	CALGDPTGANTGKLTF	CTCSAGRGGYAEQFF	09/14/22	2.5	16	15
8DNT	CAVREGAQKLVF	CASSLDLGADEQFF	07/19/23	3.18	12	14
8GOM	CASSGNTPLVF	CASTWGRASTDTQYF	03/01/23	2.78	11	15
8GON	CASSGNTPLVF	CASTWGRASTDTQYF	03/01/23	2.6	11	15
8GVB	CAVGFTGGGNKLTF	CASSDRDRVPETQYF	10/19/22	3.2	14	15
8SHI	CATDALYSGGGADGLTF	CASSYSEGEDEAFF	06/28/23	2.9	17	14
**MHC Class II**						
**PDB**	**CDR3α**	**CDR3β**	**Release date**	**Resolution**	**CDR3α length**	**CDR3β length**
7RDV	CAASDDNNNRIFF	CASGGQSNERLFF	07/27/22	2.9	13	13
7SG0	CALSGGTSYGKLTF	CASSQGQDTEAFF	02/23/22	3	14	13
7SG1	CLVGGLARDMRF	CSVALGSDTGELFF	02/23/22	3.1	12	14
7T2B	CATDKKGGATNKLIF	CASSQGGGEQYF	12/28/22	2.8	15	12
7T2C	CLVGDTGFQKLVF	CSARDPGGGGSSYEQYF	12/28/22	3.1	13	17
7Z50	CAASVRNYKYVF	CASSRQGQNTLYF	07/20/22	2.65	12	13
