# Supplementary material for: Exploring the potential of structure-based deep learning approaches for T cell receptor design
Source: PLoS Comput Biol. 2024 Sep 30;20(9):e1012489. doi: 10.1371/journal.pcbi.1012489 (PMC11466415; doi:10.1371/journal.pcbi.1012489)
Supplement: S2 Table — The table includes the experimental ΔG (in kcal/mol) for each complex and the corresponding ΔΔG (ΔGmut—ΔGwt). All experimental data was collected from ATLAS database (https://atlas.wenglab.org/). (PDF) [file pcbi.1012489.s035.pdf]

S2 Table. List of the wild-type and the corresponding mutant PDB structures of TCR:pMHC complexes that compose the benchmark for binding affinity calculations with MM/PBSA. The table includes the experimental ΔG (in kcal/mol) for each complex and the corresponding ΔΔG (ΔG_{mut} - ΔG_{wt}). All experimental data was collected from ATLAS database (`https://atlas.wenglab.org/`).

Wild-type TCR:pMHC PDB	Experimental ΔG (kcal/mol)	Mutant TCR:pMHC PDB	Experimental ΔG (kcal/mol)	Experimental ΔΔG Mut-WT (kcal/mol)
1AO7	-7.49	4FTV	-11.45	-3.96
2VLJ	-7.21	2VLR	-7.24	-0.03
3MV7	-7.72	3MV9	-7.15	0.57
3MV7	-7.72	3MV8	-6.08	1.64
2BNR	-6.13	2F53	-12.28	-6.15
2BNR	-6.13	2P5E	-14.08	-7.95
3QDG	-6.76	4L3E	-10.05	-3.29
