# Supplementary material for: Exploring the potential of structure-based deep learning approaches for T cell receptor design
Source: PLoS Comput Biol. 2024 Sep 30;20(9):e1012489. doi: 10.1371/journal.pcbi.1012489 (PMC11466415; doi:10.1371/journal.pcbi.1012489)
Supplement: S3 Table — The table includes the experimental ΔG (in kcal/mol) for both wild-type and mutant, as well as the corresponding ΔΔG (ΔGmut—ΔGwt). All experimental data was collected from ATLAS database (https://atlas.wenglab.org/). (*) The mutation numbering presented follows the same used in ATLAS database. (PDF) [file pcbi.1012489.s036.pdf]

S3 Table. List of the wild-type and the corresponding TCR mutant that composes the benchmark of modeled mutants for binding affinity calculations with MM/PBSA. The table includes the experimental ΔG (in kcal/mol) for both wild-type and mutant the corresponding ΔΔG (ΔG_{mut} - ΔG_{wt}). All experimental data was collected from ATLAS database (`https://atlas.wenglab.org/`). (*) The mutation numbering presented follows the same used in ATLAS database.

Wild-type TCR:pMHC PDB	Experimental ΔG (kcal/mol)	TCR mutations*	Experimental ΔG (kcal/mol)	Experimental ΔΔG Mut-WT (kcal/mol)
2VLJ	-7.21	S99A	-7.24	-0.03
		Q58E	-7.21	0.00
		Y101A	-6.98	0.23
		Y101F	-6.71	0.50
		S31A	-6.58	0.63
		Q34A	-6.23	0.98
		N55A	-6.08	1.13
		I53L	-5.79	1.42
		D32A	-5.63	1.58
3MV7	-7.72	Q55A	-7.15	0.57
		Q55H	-6.08	1.64
1AO7	-7.49	A99M | G100S | G101A | R102Q	-11.45	-3.96
		A99M | G100S | G101A | R102E	-11.39	-3.90
		A99M | G100S | G101A	-11.04	-3.55
		G100S | G101A | R102E	-10.94	-3.45
		A99M	-7.80	-0.31
2BNR	-6.13	Q51T | S52P | S53W | T95L | S96L | G97D | S99T | G50A | A51I | G52Q | I53T | V95L	-14.08	-7.95
		Q51P | S52F | S53W | G50S | A51G | I53M	-12.12	-5.99
		I53T | T95L | S96L | G97D | S99T | V95L	-9.67	-3.54
		Y31D | Q51T | S52P | S53W | T95L | S96L | G97D | S99T | G50A | A51I | G52Q | I53T | V95L	-6.47	-0.34
		Q50P | S51F | S52W | G49S | A50V | I52M	-12.28	-6.15
		Q50S | S51P | S52W | G49S | A50V | I52M	-11.87	-5.74
		Q50M | S51G | S52T | G49S | A50V | I52M	-11.05	-4.92
3QDG	-6.76	D26Y | L98W	-10.39	-3.63
		D26W | L98W	-10.21	-3.45
		D26Y	-8.64	-1.88
		D26W	-8.41	-1.65
		L98W	-7.55	-0.79
		A55P	-6.92	-0.16
		R27P	-6.71	0.05
		R27W	-6.25	0.51
		F100W	-5.92	0.84
		G28P	-5.76	1.00
		F100Y	-5.46	1.30
		G28Y	-5.35	1.41
