# Supplementary material for: Exploring the potential of structure-based deep learning approaches for T cell receptor design
Source: PLoS Comput Biol. 2024 Sep 30;20(9):e1012489. doi: 10.1371/journal.pcbi.1012489 (PMC11466415; doi:10.1371/journal.pcbi.1012489)
Supplement: Attachment — Submitted filename: rebuttal_response_tcr_ploscb.pdf [file pcbi.1012489.s037.pdf]

6th August 2024

Dear Editor,

We are resubmitting our manuscript entitled "**Exploring the Potential of Structure-Based Deep Learning Approaches for T cell Receptor Design**" for consideration in PLOS Computational Biology. We are grateful to the reviewers and editors for their constructive and insightful feedback, which has significantly enhanced the quality of our manuscript.

In our revised manuscript, we have meticulously addressed each of the reviewers' comments and implemented substantial changes to the original version. Key modifications include:

- Structural analysis of TCR test cases, featuring TCR contacts with pMHC and relative solvent accessibility of TCR variable domain positions, enhancing the discussion throughout the manuscript.
- Addition of sequence variability analysis for TCR variable domain regions.
- Direct comparison of sequence recovery between ProteinMPNN and ESM-IF1.
- Assessment of CDR3 generation probability using the OLGA algorithm.
- Inclusion and analysis of two new design scenarios: design of all CDR positions and design of the entire TCR variable domain.
- Analysis of sequence recovery in the presence and absence of the pMHC context at hotspot positions.
- Modeling, confidence scoring with TCRModel2, RMSD analysis, and Rosetta scoring of designs generated by ProteinMPNN and ESM-IF1 for all CDR positions, including random CDRs.
- Sequence recovery analysis of modeled structures in addition to solved structures for both ProteinMPNN and ESM-IF1.
- Expansion of the MM/PBSA benchmark to include 35 new modeled TCR mutants, all analyzed through molecular dynamics simulations and MM/PBSA calculations.
- Revising the color scheme in figures for improved clarity.

Modifications are tracked in the revised version of the manuscript. Our responses to the reviewer's comments are highlighted in blue on the following pages.

We are confident that our manuscript will be of great interest to the readers of PLOS Computational Biology, and we trust that the revisions meet your expectations for publication.

Thank you for your time and consideration.

Your sincerely,

Helder V. Ribeiro-Filho, PhD & Paulo S. Lopes-de-Oliveira, PhD
(helder.ribeiro@lnbio.cnpem.br) (paulo.oliveira@lnbio.cnpem.br)

Responses to reviewers

Reviewer #1:

In this work, the authors evaluate different inverse folding algorithms for TCR-pMHC binding design. This is great and thorough work. i have a few comments.

We appreciate the reviewer's feedback and thank the reviewer for thoroughly reviewing the manuscript.

- you use sequence recovery as a main metric. Given that one wants diversity in the designed sequences, why is sequence recovery, which measures the conservation of native positions, a good metric? can you explain this better in the manuscript?

We appreciate the opportunity to clarify this point. We used the recovery of native TCR sequences as a metric to evaluate our designs because it estimates how closely the design sequences situate with known binder sequences. By utilizing the reference TCR structure dataset, we gain insights into the typical features of TCR binders to given pMHCs and through inverse folding based on fixed TCR backbones, we can assess how effectively these methods replicate natural and true binder interfaces. Demonstrating that these methods can generate TCR interfaces similar to native ones—especially in this specific interface involving loops and characterized by low affinity—is valuable. This knowledge enhances our confidence in designing pMHC binders for which no native TCR is known. Importantly, sequence recovery is a widely used metric in various protein design methods (DOI: 10.1038/s41467-024-50571-y;10.1002/prot.25489; 10.1126/science.add2187; 10.1101/2022.04.10.487779; 10.1093/bioinformatics/btae037; 10.1016/j.csbj.2022.11.014), allowing us to compare our results with those from the design of general proteins, as we discussed in the original version of the Discussion section (line 382).

In the sequence recovery analyses (Figures 1-4), it is possible to observe that while some designed sequences exhibit high recovery, the sampling temperature employed also allows for significant diversity, including the generation of sequences with low recovery. As previously noted in the original discussion section (line 395), while sequence recovery is a valuable metric, sequences with low recovery—indicating greater diversity—can potentially be better binders. For this reason, in this study, we thoroughly evaluated the diversity of designs generated using native agonistic structural methods, including computing confidence modeling scores, interface scores, and binding affinity. These evaluation approaches were planned to assess the designs independently of their similarity to known native binders.

We have included a paragraph in the Introduction section (line 98 in the version with tracked changes) to clarify the importance of sequence recovery in our study, while also highlighting the limitations of the sequence recovery metric and the use of alternative

structure-based methods for overcoming these limitations. We have also revised sentences in the Results section to avoid any misinterpretation that sequence recovery is the most important metric in our study.

- can you also use immunebuilder (in additon to tcrmodeler2) to investigate the designs structurally?

From our understanding, immunebuilder (TCRBuilder2+ as its latest version for TCR modeling) will only be able to model unbound TCR structures. While this task is relevant, our study aimed to model the structure of the TCR designs in complex with the corresponding pMHC. This approach helps us determine if the modeling supports the TCR binding. Even when modeling unbound states, interpreting the conformational space of CDR loops, especially CDR3 loops, is limited due to their structural diversity, as shown in the clustering analysis from the TCRBuilder2+ manuscript [DOI: 10.1101/2024.05.20.594940].

Additionally, as indicated in the results from TCRBuilder2+ [DOI: 10.1101/2024.05.20.594940, table 1], AF-Multimer, whose trained weights are utilized in TCRModel2 [Yin et al, 2023], either outperforms or at least provides a comparable accuracy (in terms of CDR RMSD) in comparison to TCRBuilder2+. A recent independent benchmark study also highlighted the accuracy of TCRModel2 in modeling TCR:pMHC complexes in comparison to other modeling approaches [DOI: 10.1016/j.csbj.2024.06.028].

To clarify this topic, we have added a sentence in Discussion Section 3 (line 593 in the version with tracked changes) about future investigation of unbound states of TCR designs.

- can you also run your designed TCRs through OLGA and test if they are actually native-like TCRs? If the sequence pgen is very low, then the designs are likely not very natural and thus not useful for therapeutics application (immunogenic).

We appreciate the reviewer's suggestion, which has provided valuable insight into our study. As suggested by the reviewer, we utilized OLGA to analyze the designed CDR3 sequences from ProteinMPNN and ESM-IF1 across two different design scenarios: the design of only CDR3 interface positions, and the design of all CDR3 positions.

We have incorporated the OLGA results into section 2.2 (line 293 in the version with tracked changes), updated Figure 3 accordingly, and included a discussion about this topic in Discussion section 3. The corresponding methods have been added to Methods section 4.

Reviewer #2:

In this manuscript, Ribeiro-Filho et al. explore the application of inverse folding models (ProteinMPNN and ESM-IF1) to the fixed-backbone CDR sequence design of TCRs. They evaluate the models' performance via sequence recovery, refolding, and energetic scoring (using Rosetta and MM/PBSA calculations). The authors demonstrate that the tested inverse folding methods can recapitulate a substantial portion of native sequences and produce designs with equal, and in some cases greater, predicted binding affinity to the target peptide-MHC complex than the starting TCR sequence.

This work provides value to the field by examining the performance of inverse folding models on TCRs, which, as the authors point out, are underexplored in the protein design space. However, the manuscript is currently somewhat limited in novelty and scope. The authors use existing, well-established inverse folding models and evaluation methods. Additionally, the sequence design analysis is primarily limited to CDR3 loops. The paper would be strengthened by addressing the following points.

We appreciate the reviewers' feedback on the value of our study and their careful review of our manuscript. The comments and questions from the reviewers led to several additional analyses, aiming to enhance the quality of our work. We wish to clarify that the novelty of our study lies in a detailed and comprehensive evaluation of the deep-learning based inverse folding methods for designing TCR. ProteinMPNN and ESM-IF1 are well-established for general protein design tasks, especially for monomeric proteins. This makes it essential and beneficial for the scientific community engaged in this field to expand the understanding of their applicability in designing protein interfaces, especially the unique TCR interface as explored in our study. We employed a comprehensive set of sequence- and structure-based evaluation metrics, incorporating both recently developed deep-learning modeling methods (TCRModel2) and classical physics-based methods (molecular dynamics simulations with MM/PBSA calculations), which were rigorously benchmarked for this specific task. As suggested by the reviewer, as one of the modifications in the revised version, we expanded the analysis beyond the CDR3 loops.

Major comments

The manuscript should make a stronger case for the intended application(s) and the applicability (or lack thereof) of the inverse folding models. As it stands, only the re-design of CDR (primarily CDR3) loops for structurally-resolved TCR-pMHC complexes is investigated. This may only cover a fraction of real-world use cases for TCR inverse folding. Extending the

analyses to some of the following suggestions would provide a more thorough understanding of the models' applications and limitations:

- What are the inverse folding models stronger or weaker at, respectively and/or in general? A more detailed investigation of failure modes would be helpful.

We thank the reviewer for pointing this out. In the original manuscript (Results section 2.7), we examined the inability of ProteinMPNN and ESM-IF1 to generate designs with similar or higher affinity compared to the 7pdw test case. This included performing energy decomposition for this test case and discussing the specific challenges associated with it. However, we agree that a deeper analysis, particularly comparing ProteinMPNN and ESM-IF1, would enhance the quality of the manuscript. Therefore, we have included the following analyses in the revised version of the manuscript:

- **Sequence recovery comparison between ProteinMPNN and ESM-IF1:**

We conducted a correlation analysis between the maximum sequence recoveries achieved by ProteinMPNN and ESM-IF1 to determine if both inverse folding methods might be redundant in terms of design performance. This analysis also provides an improved view of when one design method outperforms the other.

We have added the results of this analysis into Results section 2.1 (line 171 in the version with tracked changes), Discussion Section 3 (line 556 in the version with tracked changes), and a scatter plot as a new Supplementary Figure S7.

- **Dependency of solvent exposure on inverse folding method performance accessed by Relative Solvent Accessibility (RSA)**

We conducted an analysis to assess the impact of solvent exposure on the performance of ProteinMPNN and ESM-IF1 regarding sequence recovery at designed CDR3 interface positions. In addition to the general assessment of the impact of buried positions on sequence recovery, as presented in Figure 4, we also analyzed the relative solvent accessibility (RSA) for each designed amino acid. We examined whether the original amino acid at those positions was recovered. Although this difference was significant—as also shown in Figure 4 for ProteinMPNN and in the new analysis included—the difference in RSA ratios is minimal (median of 0.05 for recovered versus 0.09 for not recovered with ProteinMPNN, and 0.06 for recovered versus 0.09 for not recovered with ESM-IF1). This suggests that generally, there is no clear difficulty for both ProteinMPNN and ESM-IF1 in recovering buried or more solvent-exposed positions at the CDR3 interface with the pMHC.

We have included this new analysis in the new Supplementary Figure S14, and detailed it in Results Section 2.3 (line 323 in the version with tracked changes) and Discussion section 3 as well.

- **Analysis of sequence recovery in the presence or absence of the target pMHC at hotspot positions**

To enhance the comparison between ProteinMPNN and ESM-IF1 regarding the recovery of native sequences with and without the target pMHC, which also addresses a minor reviewer question about the last sentence of the orignal section 2.4, we replicated the analysis shown in the original Figure 5. However, this time we focused solely on recovery at CDR3 hotspot positions. In this refined analysis, we still observed test cases with the same maximum sequence recovery, whether or not the pMHC was present. This finding may indicate that the reason for maintaining sequence recovery in the absence of pMHC is not fully explained by recovery at non-essential binding positions. This analysis was added as Supplementary Figure S15.

We have added a new Figure 5, which now illustrates the differences between the maximum sequence recovery with and without the presence of the pMHC, to evaluate which inverse folding method's recovery is more significantly affected by the removal of the pMHC context. Generally, we did not observe any significant differences. However, the magnitude of the difference was higher for both ProteinMPNN and ESM-IF when considering only hotspot positions (Figure S15), highlighting the relevance of the pMHC interface in the designing process. It is also crucial to note that the maintenance of sequence recovery, whether the pMHC is present or absent, observed in some test cases, does not remain consistent across the inverse folding methods. The Pearson correlation between the differences in sequence recovery for ProteinMPNN and ESM-IF is only 0.22 (considering only hotspot positions or not). Thus, despite the impact on sequence recovery caused by removing the pMHC context being generally similar for ProteinMPNN and ESM-IF, the extent of this impact across test cases is not highly correlated between these methods. This suggests that the influence of pMHC removal on sequence recovery for each test case is not solely determined by the structure itself, but that the methods may behave differently.

The results from these analyses have been included in Section 2.4 of the Results (line 360 in the version with tracked changes).

- Expand sequence design beyond the CDR3. Given that the randomly designed CDR3 sequences had similar RMSDs to the designed sequences and TCRs with the same CDR3 sequence can bind different pMHC complexes (as mentioned in section 4.1), an expanded analysis of sequence design for other TCR regions could provide more insights into model performance. Additionally, this would open the door to further important evaluations, such as whether generated sequences map back to known V/J genes (and, if so, whether these genes are associated with the native loop canonical forms).

We thank the reviewer for the suggestion. Following the reviewer's suggestion, and in addition to the original analyses of designs for CDR3 interface positions, all CDR3 positions,

and CDR1, CDR2, and CDR3 interface positions, we have now expanded our design analyses to include all positions of CDR1, CDR2, and CDR3. We expanded the evaluation of the designs generated in this new design scenario beyond the sequence recovery and included this design scenario into the following analysis:

- Structure modeling with TCRModel2: Despite the increased number of designed positions, designs from both ProteinMPNN and ESM-IF1 were still able to achieve high model confidence scores and low deviations from the native structure. These results have been included in Section 2.5 of the Results (line 408 in the version with tracked changes) and a new Supplementary Figure S18 has been added.
- Rosetta scoring: Similarly to the model confidence, Rosetta dG_separated and total_scores indicated that designs of all CDR positions can achieve similar or better scores compared to native TCRs, more clearly contrasting with random CDRs. These results have been included in Section 2.6 of the Results (line 453 in the version with tracked changes), and a new Supplementary Figure S21 has been added.
- Binding affinity: We estimated the binding energies of designs involving all CDR positions for the 7pdw and 7pbe test cases to assess whether expanding the design interface to include MHC interactions could enhance affinity, which was not achieved with the CDR3 interface designs. These results are detailed in Results section 2.7 (line 506 in the version with tracked changes), and a new Supplementary Figure S27 has been added for illustration.

In addition to focusing on the TCR interface design, this study also expanded to designing the full TCR variable domain, conditioned to the pMHC, to better understand the challenges associated with designing positions other than CDRs. We analyzed the per-position sequence recovery for both TCRα and TCRβ, detailing the findings in section 2.1 and adding new Supplementary Figure S12. Highly conserved positions within the TCRs showed the highest sequence recoveries. This trend was consistent between ProteinMPNN and ESM-IF1 designs and was not strongly correlated with solvent accessibility.

- Apply the inverse folding approaches to predicted structure inputs. In real design use cases, one may not always have a solved structure of the starting complex. Are the models still able to achieve strong performance without an experimentally solved structure?

We thank the reviewer for this relevant suggestion, which indeed provided significant insights into alternative design scenarios. To evaluate the performance of the inverse folding methods using predicted backbone structures, we designed the TCR CDR3 interface positions based on the TCRModel2 native structures described in Section 2.5.

We calculated the maximum sequence recovery for the 3D model of each test case and compared it with the sequence recoveries from the X-ray crystallographic structures originally used in this study. Generally, the inverse folding methods employing 3D models still obtained a

similar magnitude of maximum sequence recovery as those obtained from X-ray structures. The most significant differences were associated with higher deviations in CDR3 backbone positions.

We have added a paragraph detailing these results in Results Section 2.5 (line 422 in the version with tracked changes) and included a new Supplementary Figure S19. This figure not only displays bar plots of maximum sequence recovery but also a scatter plot that shows the correlation between the differences in sequence recovery observed between 3D models and X-ray structures and the CDR3 backbone RMSD, providing explanatory context for these results.

- Polyreactivity: although the authors state this is beyond the scope of their manuscript, it would be valuable to understand, for example, if the inverse folding model suggestions increase affinity for the MHC rather than the peptide and thereby increase the risk of polyreactivity.

We appreciate the reviewer's interest in this topic. Since the inverse folding methods evaluated in this study utilize fixed backbone structures, the specificity during the design process is more related to the rational selection of appropriate design positions rather than the model suggesting amino acids per se. For this reason, in this study we focused the application of inverse folding methods on CDR3 interface positions, which is the region of the TCR that most extensively contacts the peptide. Thus, improvements in affinity observed should be associated with that peptide interface region.

In future studies, we aim to explore in detail the application of inverse folding methods and their modifications as well in a case-specific cross-reactivity context. A notable application would be in the TCR positive-negative balanced design [doi: 10.1016/j.ymthe.2018.12.010], where one could select amino acids that do not have the highest probability for positions of the TCR that only contact the MHC, while selecting amino acids with the highest probability for TCR positions that contact the peptide.

We have added Supplementary Figure S1, which illustrates the contacts between the TCR regions and the pMHC for all MHC-I and MHC-II test cases analyzed. This analysis is helps to emphasize the importance of CDR3 contacts with the peptide for specificity and to substantiate statements made throughout the manuscript.

- The MM-PBSA affinity calculation benchmark gives a helpful understanding of the potential accuracy of this approach. However, this accuracy is not directly applicable to the use case in this manuscript: for the benchmark, only cases with a solved structure of the wild-type and mutant were included; for the assessment of inverse folding designs, the mutant structures were modeled. The MM-PBSA benchmark should be updated/extended to include cases with modeled mutant structures to provide a relevant quantification of this protocol’s accuracy.

We thank the reviewer for the suggestions. Following the reviewer's suggestion, we expanded our MM/PBSA benchmark to include mutants of the same original five native TCRs that have experimentally determined ΔG but lack solved structures. These mutants were modeled from the solved structure of the corresponding native TCR using the same protocol employed for modeling the designs. A total of 35 mutants were included in this extended benchmark.

In general, although the correlation between calculated and experimental ΔΔG using modeled structures is reduced compared to that obtained from solved structures, it remains high (Pearson correlation of 0.74) and useful for our evaluation purposes. This correlation from modeled mutant structures compares favorably with that reported in a more restrictive benchmark by Cream et al. 2022 [doi: 10.1021/acs.jcim.1c00765]. It is also on par with or exceeds those achieved by classical or optimized/trained energy functions for TCR binding affinity prediction [doi: 10.1371/journal.pcbi.1003478, 10.1093/protein/gzw050], with the added advantage of not requiring training or fine-tuning on TCR data, thereby avoiding issues with generalizability.

Additionally, we assessed the performance of the calculated ΔΔG by MM/PBSA using modeled structures to discriminate improvements in ΔΔG greater than 0.5 kcal/mol or 2 kcal/mol resulting from mutations. Using a confusion matrix, we found balanced accuracy scores of 0.79 and 0.85, respectively, for these two ΔΔG improvement ranges.

We have integrated these results into a new Supplementary Figure S22 and presented them in Results Section 2.7 (line 474 in the version with tracked changes).

- It would be useful to provide an understanding of natural sequence variability in TCRs across different portions of the structure (down to single-position resolution for CDR3 at least). This will give more insights into the relative challenges of sequence recovery tasks.

We appreciate the reviewer's suggestion and have added Supplementary Figure S3, which presents sequence logos illustrating the variability of TCR frameworks and CDR1 and CDR2 regions encoded by TRAV and TRBV genes. The figure also includes the TCR J region encoded by the TRAJ and TRBJ genes, along with the variability of CDR3α and β from sequenced TCRs with known antigen specificity.

It is important to note that the designs (particularly CDR3) obtained from the structure-based inverse folding methods evaluated in this study are conditioned to the CDR3 backbone structure and the interactions with the corresponding target pMHC structure. Thus, while we observe high variability in the central part of the CDR3 (as shown in the figure below), given the structural constraints, the expected and acceptable variability would be much lower than that seen in a combined set of general natural TCRs.

This analysis also helped to support several statements made in the manuscript.

Minor comments

- It would be helpful if the authors more clearly stated/explained the following: In section 2.1, it should be more clearly stated that the antigen is provided as context for the models. Additionally, the authors should explain whether the rest of the TCR sequence is given to the model as context and whether the CDR sequences were sampled autoregressively or in a single forward pass.

In the revised manuscript, we detailed in Results section 2.1 (line 142 in the version with tracked changes) that the pMHC and non-designed TCR residues were provided as context during the design process. Regarding the decoding method, since it is inherent to the method used, we have provided detailed information in Methods section 4.2. While ESM-IF1 employs a canonical autoregressive approach to sequence sampling, ProteinMPNN uses an order-agnostic autoregressive model, where the decoding order is randomly determined.

- In Figure 1B, a sequence recovery value of exactly 50% appears to be overrepresented. Is there an explanation for this?

The overrepresentation of a 50% sequence recovery value in Figures 1B and 4 arises from the discrete nature of the dataset concerning sequence recovery percentages. Although presented as a continuous variable, sequence recovery data is inherently discrete, being calculated based on the integer count of designed amino acids (Equation 1). This discreteness leads to noticeable "lines" at specific values, such as 50%, and also at other values like 20% and 33.3%. For instance, in the CDR3 interface design scenario, 60% of the test cases have an even number of designed residues, making a 50% recovery rate more likely. Examples include sequences with 3/6, 4/8, 5/10, 6/12, and 7/14 recovered residues, all yielding a 50% recovery rate (57 occurrences in the dataset).

- What percent of model designs are redundant?

This percentage is represented as "Uniqueness" in Figure S2. We have detailed this information in the Methods Section 5, under the subsection "Design quality assessment at sequence level," specifically in 4.4.

- In the Figure 4 legend, the buried AAs are defined as a subset of the hotspot AAs, but this does not seem to match section 4.5.

The reviewer is correct; we have made the appropriate corrections in the legend of Figure 4.

- In section 2.5, the TCRModel2 confidence metric should be explained, as this is a major underpinning of the analyses presented in this section. Additionally, it should be clearly specified which confidence metric is used. (I presume the linear combination of pTM and ipTM, as this is defined as 'model confidence' in the TCRModel2 publication, but the paper also states that pLDDT, pTM, and ipTM confidence metrics are provided).

The confidence score is a weighted combination of ipTM and pTM (ipTM*0.8 and pTM*0.2, same as described in AlphaFold-Multimer article [doi: 10.1101/2021.10.04.463034]. We have included this information in Results Section 2.5 (line 377 in the version with tracked changes) and Method subsection 4.6 (line 768 in the version with tracked changes).

- Also in section 2.5, the authors should state and comment on the results in Figure S10, that random CDR3 sequences achieve similar RMSDs to designed sequences.

In the revised version of the manuscript, we have addressed and included the observation of random CDR3 sequences achieving similar RMSD to designed sequences in Results Section 2.5 (line 403 in the version with tracked changes).

- Could the authors elaborate on how the same sequence can achieve different ProteinMPNN scores, as stated in the Figure S5 caption? Would the log-probability outputs not be fixed for a given structure input (and used to sample/score multiple sequence outputs)?

In ProteinMPNN outputs, the same sequences can appear with different ProteinMPNN scores. This phenomenon is related to the order-agnostic autoregressive model used in ProteinMPNN, where the decoding order is randomly determined. Therefore, depending on the randomization during decoding, it is possible to obtain the same decoded sequences but with different negative average log-probabilities.

- The following text could be updated: In the abstract, I would suggest that immunotherapeutic applications of design approaches are "underexplored" rather than "unexplored".

We replaced 'unexplored' with 'underexplored' in the Abstract (line 6 in the version with tracked changes).

- In the introduction, I believe that it is not fully accurate to say that ProteinMPNN designs "entirely novel proteins", as the backbone is not novel (fixed). Perhaps this could be amended to "novel sequences".

We replaced 'novel proteins' with 'novel protein sequences' in the Introduction (line 85 in the version with tracked changes).

- In section 2.1, a more likely explanation for the poorer performance on MHC-II- than MHC-I-binding TCRs is the lower number of training structures, not test structures.

There does not seem to be any evidence to support that the test cases included here would be more challenging than other potential MHC-II test cases.

Given the limited number of MHC-II test cases (6 in this study), we originally suspected that the observed reduced sequence recovery of MHC-II test cases might simply reflect a statistical sampling bias—that is, we analyzed test cases with lower sequence recovery by chance. To test this hypothesis, in the revised version we computed the cumulative probabilities of the MHC-II maximum sequence recovery averages for ProteinMPNN, ESM-IF1, and Rosetta Design under the distributions of MHC-I maximum sequence recovery averages, constructed by sampling averages from 6 random test cases (the same number as MHC-II test cases) 1000 times. We obtained two-tailed p-values of 0.012, 0.008, and 0.023 for ProteinMPNN, ESM-IF1, and Rosetta Design, respectively. These results indicate that the maximum averages observed for MHC-II test cases significantly differ from those observed for MHC-I.

We have enhanced the explanation in Section 2.1, incorporating the alternative possibility suggested by the reviewer regarding the lower number of training structures available for MHC-II test cases (line 179 in the version with tracked changes).

- Towards the end of section 2.1, "interestingly" should be removed. As the authors point out, sequence recovery is expected to be higher across all rather than just interface CDR3 positions due to greater sequence conservation.

We removed 'Interestingly' from the sentence in section 2.1 (line 233 in the version with tracked changes).

- The last sentence of section 2.4 would require more supporting evidence. The data could alternatively be interpreted as the sequence recovery remaining unchanged due to the relatively low/moderate recovery success; the models could, for example, only be recovering non-interface or non-essential binding positions.

We thank the reviewer for pointing this out. We do not believe that low/moderate recovery success is the reason for unchanged recovery without the pMHC context, as the two cases mentioned in the original manuscript (7nme for ProteinMPNN and 6zkw for ESM-IF1) both exhibit high levels of maximum sequence recovery (greater than 60%) with unchanged recovery in the absence of pMHC. Following the reviewer's suggestion, we evaluated the effect of pMHC removal on the recovery of only essential (hotspot) positions. We observed a more substantial reduction in sequence recovery without the pMHC context, highlighting the importance of the specific pMHC interface in the design process. However, even when considering only hotspot positions, we still encountered cases with the same sequence recovery with or without pMHC. This suggests the involvement of other factors in this phenomenon. We have incorporated these new findings into Results section 2.4 (line 360 in the version with tracked changes) and have enhanced the interpretation of results in the revised version of the manuscript.

- The ESM-IF1 inverse folding model should be referred to as “ESM-IF1” rather than “ESM-IF”.

We have updated 'ESM-IF' to 'ESM-IF1' throughout the manuscript, including in all figures.

- The figure colors should be kept consistent throughout (e.g., always green for native).

We have reviewed and updated the color scheme of the figures where appropriate. In the manuscript's revised version, we consistently used purple for ProteinMPNN analysis and results, red for ESM-IF1, blue for Rosetta Design, and green for Native. (note: organization of jitter points in newly generated figures may slightly differ on the x-axis due to the randomness of the ggplot jitter algorithm when replotting in modified plots, but the y-values remain as previously)."

- In Figure 2B, one CDR3b appears to be in a very different place than the other CDRs. Could the authors comment on this?

This CDR3β is from the PDB structure 7l1d. The TCR from this structure features a long CDR3β, which adopts a distinct conformation compared to other analyzed CDR3βs. We have highlighted this information in the legend of Figure 2.

- The amino acid groupings in section 4.4 are missing P and T. Additionally, it would be helpful to keep the groupings consistent with Figure 3.

We corrected the grouping by adding the missing amino acids and decided to maintain consistency by using BLOSUM62 clustering into 6 groups ([A, G, S], [C], [D, E, P, T], [Q, N, H, R, K], [I, L, M, V], [F, Y, W]) throughout the manuscript. This included updates to Figure 3 in Section 2.2 and the similarity analysis in Figure 4, Section 2.3. In the revised version, these sections also maintain consistency with generating random sequences by selecting dissimilar amino acids based on the amino acid grouping. Importantly, these updates did not significantly alter the results or their interpretations.

- Figure S12B should be square (equal x- and y-axes) to show that, although the correlation is strong, the calculated and experimental dG results are not on exactly the same scale.

In the new Supplementary Figure S22, we have scaled the axes.

- The caption of Figure S4 only explains A-B, but the figure includes panels A-D.

We apologize for the confusion and have provided the correct legend in the revised version of the supplementary material (Figure S5)
