# Supplementary material for: Exploring the potential of structure-based deep learning approaches for T cell receptor design
Source: PLoS Comput Biol. 2024 Sep 30;20(9):e1012489. doi: 10.1371/journal.pcbi.1012489 (PMC11466415; doi:10.1371/journal.pcbi.1012489)
Supplement: Attachment — Submitted filename: rebuttal_response_tcr_ploscb.pdf [file pcbi.1012489.s038.pdf]

Brazilian Biosciences National Laboratory

LNBio

CNPEM
Brazilian Center for Research in Energy and Materials

5th September 2024

Dear Editor,

We are submitting the second revised version of our manuscript entitled "**Exploring the Potential of Structure-Based Deep Learning Approaches for T cell Receptor Design**" for consideration in PLOS Computational Biology. We are grateful to the reviewers and editors for their constructive and insightful feedback, which has significantly enhanced the quality of our manuscript.

In this revised version of the manuscript, we have addressed the minor analyses, text clarifications and the text and figure changes suggested by reviewer #2. The data in the Zenodo repository has also been updated to reflect the additional analyses performed.

Modifications in this second revised version of the manuscript are tracked in comparison to the first revised version. Our responses to the reviewer's comments are highlighted in blue on the following pages.

We are confident that our manuscript will be of great interest to the readers of PLOS Computational Biology, and we trust that the revisions meet your expectations for publication.

Thank you for your time and consideration.

Your sincerely,

Helder V. Ribeiro-Filho, PhD & Paulo S. Lopes-de-Oliveira, PhD
(helder.ribeiro@lnbio.cnpem.br) (paulo.oliveira@lnbio.cnpem.br)

Responses to reviewers

Reviewer #1:

The authors have addressed all of my comments.

We are pleased to have been able to address all of the reviewer's comments.

Reviewer #2:

The authors have made significant revisions to the manuscript, addressing the feedback from the reviewer comments and strengthening the paper. The added analyses provide a clearer understanding of the strengths, limitations and functionalities of inverse folding models for TCR sequence design. Additionally, the updates to the Discussion section highlight interesting points and areas for future exploration.

I have a few minor comments arising from the changes. The line numbers are indicated for the version with tracked changes.

We appreciate the reviewer's recognition of our efforts to strengthen the manuscript and are grateful for the constructive suggestions and comments provided.

Minor analysis

• Line 171, Fig S7: Are there any unifying features for the cases which ESM-IF1 or ProteinMPNN, respectively, performed better on? (i.e. those which are above vs. below y=x in Fig S7)

To provide further interpretability of the results presented in Fig. S7, we decided to explore deeper sequence and structural features that might account for the higher maximum sequence recovery achieved by either ESM-IF1 or ProteinMPNN in certain test cases. The new analysis has been incorporated into a new supplementary figure S7. We divided the test cases into two groups: one where ESM-IF1 achieved higher maximum recovery, and another where ProteinMPNN did. To minimize noise in the analysis, we only included test cases with at least a 10% improvement in recovery when comparing the methods. This resulted in 8 test cases in the ProteinMPNN group (6zkw, 7l1d, 7na5, 7pbc, 7rrg, 8cx4, 8gon and 8gvb) and 13 cases in the ESM-IF1 group (7dzm, 7n2n, 7n2o, 7n2p, 7n2q, 7n2r, 7n2s, 7ndq, 7nmg, 7ow6, 7pbe, 7phr and 8dnt).

We analyzed the per-position frequency of native amino acids in the CDR3s (Fig. S7C). Aside from a slight increase in the preference of certain amino acids such as at positions 110 and 111 of CDR3β for ESM-IF1, no clear pattern emerged that could explain the differences in maximum sequence recovery. The length of CDR3s in both groups was not

significantly different (Fig. S7D), nor were the buried surface area and the shape complementarity of the TCR interaction interface (Fig. S7E) (all metrics obtained from TCR3d - https://tcr3d.ibbr.umd.edu/).

To assess whether certain amino acids might be challenging to recover regardless of their positions in the sequence, we estimated the frequency of native amino acids recovered by ProteinMPNN but not by ESM-IF1 in the same test case, and vice versa (Fig. S7F). In this analysis, we observed that among the native amino acids recovered by ProteinMPNN but not by ESM-IF1, most were serine and threonine, suggesting that these amino acids are more easily recovered by ProteinMPNN. Conversely, aspartic acid and histidine were more easily recovered by ESM-IF1 compared to ProteinMPNN. These biases are also reflected in the frequency of substitutions of amino acids shown in Figure 3. However, this may not be the sole factor contributing to the differences in maximum recovery. For instance, when evaluating the 7n2p case, which showed the largest difference (71% recovery by ESM-IF1 and 28% recovery by ProteinMPNN), only about half of the native amino acids that presented preferences for ESM-IF1 recovery (phenylalanine, alanine, and aspartic acid) were present in the 7n2p designed positions (native non-contiguous amino acid sequence FATYSTD encompassing CDR3α and CDR3β). Also, the balance between recovery from CDR3α or CDR3β is maintained in ProteinMPNN and ESM-IF1. From the positions recovered by ProteinMPNN and not recovered by ESM-IF1, 40% were from CDR3α and 60% were from CDR3β These percentages were maintained in positions recovered by ESM-IF1 but not by ProteinMPNN, indicating no bias towards the TCR chains that explain the recovery differences.

Since both ProteinMPNN and ESM-IF1, as graph-based neural networks, were trained to consider the amino acid environment, we questioned whether positions recovered by ProteinMPNN but not by ESM-IF1, and vice versa, might have different distributions of surrounding amino acids (considering backbone atom distances of 10 Å). However, we did not find any substantial difference in the distribution of amino acids surrounding the designed positions that could explain improved recovery by either of the evaluated methods (Fig. S7G).

In conclusion, the difference in maximum sequence recovery may still result from features not analyzed here. However, it is possible that these differences might not directly arise from the structural characteristics themselves but rather from the sequences or structures observed during model training. Additionally, the improvement in maximum sequence recovery for test cases where ProteinMPNN outperformed ESM-IF1 was only 12% on average, making it even more challenging to identify a clear feature contributing to ProteinMPNN performance.

We have incorporated these new analyses in the Supplementary Figure S7 and in Results section (line 172 in the new revised version with tracked changes).

• Fig 5: Could you include a figure and statistical analysis similar to Fig 5B for the information portrayed in Fig 5A? (i.e. Fig 5B but with 'maximum sequence recovery' rather than 'maximum sequence recovery difference')

We have included in Figure 5 (and also in Figure S15, which contains the same analyses but focuses only on hotspot positions) a maximum sequence recovery plot along with the statistical analysis.

• Line 512, Fig S27: Can you identify any explanations (e.g., specific sequence changes) for why designing all CDRs substantially improved binding affinity as compared to designing only CDR3 for 7pbe?

To understand why designing all CDRs improved the binding affinity compared to designing only CDR3 for the 7pbe test case, we performed a per-residue decomposition of the binding free energy of the native 7pbe structure, as shown in the new Figure S28. This decomposition can indicate which TCR residues contribute positively to binding with the pMHC and which contribute negatively.

Our analysis of the native 7pbe structure identified residues in non-CDR3 regions, such as R40 in CDR1β, as contributing negatively to binding (Figure S28A). In this case, this arginine is close to another arginine from the peptide (R5) potentially generating repulsive forces (Figure S28B). The decomposition also highlighted other residues, such as N27 and A30 in CDR1α, S69 in CDR2α, and N60 in CDR2β, which neither contributed positively nor negatively to binding. These findings suggest that changes in these residues in the design process could enhance the chance to improve binding affinity.

By decomposing the binding free energy of the design that achieved higher affinity than the native structure (ESM-IF1 design #4), we found that ESM-IF1 improved the energy at CDR1β R40 by replacing arginine with glutamic acid (Figure S28B). Another significant substitution that contributed to the improved affinity was the replacement of S69, a mostly solvent exposed serine in CDR2α, with aspartic acid, enabling the anchoring of an arginine from the MHC—a binding interaction that was not possible with the native serine. Of course, these are only specific changes that must be interpreted in combination with all other counterbalancing modifications (including those that contributed negatively) to fully interpret the binding affinity.

In conclusion, expanding the design beyond the CDR3s may increase the chance of energetically improving TCR positions, particularly those that do not natively contribute positively to binding. Additionally, increasing the number of designed positions could facilitate cooperative residue changes, optimizing the local environment through multiple coordinated amino acid modifications. We plan to thoroughly explore the explainability of inverse folding methods and guide optimized designs using free energy calculations in a future study.

We have included this new analysis in the Results section (line 531 in the revised version with tracked changes) and the Discussion section (line 639 in the revised version with tracked changes). The new figure from this analysis has been added as Supplementary Figure S28.

Text – clarification

• Line 352: Could this new text be rephrased for clarity? The statements in lines 349 and 352 seem contradictory at a first glance (the differences being analyzed in these sentences – with vs. without the pMHC; ProteinMPNN vs. ESM-IF1 – could be explained more clearly). The sentences in lines 354-357 also appear contradictory; these concepts are more clearly explained in the response to reviewers comment.

We thank the reviewer for pointing this out. We have rephrased the sentences for clarity and decided to replace the boxplot of differences (Fig. 5B) with a scatter plot that illustrates the correlation between the differences in maximum sequence recovery upon pMHC removal for ProteinMPNN and ESM-IF1 (line 357 in the new revised version with tracked changes).

Text – minor changes

• Line 24: The phrase "trained on" in "this study explores whether computational methods, trained on deep learning architectures such as ProteinMPNN and ESM-IF1" is misleading and should be amended to clarify that ProteinMPNN and ESM-IF1 are directly used (as opposed to new methods trained/based on these architectures).

We have revised this sentence accordingly (line 23 in the new revised version with tracked changes).

• Line 336: It would be helpful if more information was directly provided in the Results section about the method for identifying hotspots (using Rosetta, 0.5 kcal/mol cutoff) and the relevant methods section (4.7) was referenced.

We have included the information about Rosetta and the cutoff used in the Results section, and we have also referenced the Methods section (line 338 in the new revised version with tracked changes)."

• A few typos are included and should be fixed, e.g., "residues" to "resides" in line 39 and "10-10" to "10^-10" in line 296.

We have corrected the typos accordingly (lines 38 and 300 in the revised version with tracked changes).

Figures – minor changes

• Figure S3C – The sequence position numbers are absent from panel C (included in A, B and D).

We appreciate the reviewer's observation. We have included the sequence position numbers for the panel of TRAJ and TRBJ sequences and adjusted the panel order to more clearly reflect the sequence of VDJ recombination.
